# Often *in silico*, rarely *in vivo*: characterizing endemic plant-associated microbes for system-appropriate biofertilizers

**DOI:** 10.3389/fmicb.2025.1568162

**Published:** 2025-04-28

**Authors:** Holly Hone, Tongda Li, Jatinder Kaur, Jennifer L. Wood, Timothy Sawbridge

**Affiliations:** ^1^Agriculture Victoria, AgriBio, Centre for AgriBioscience, Bundoora, VIC, Australia; ^2^DairyBio, AgriBio, Centre for AgriBioscience, Bundoora, VIC, Australia; ^3^School of Applied Systems Biology, La Trobe University, Bundoora, VIC, Australia; ^4^Department of Microbiology, Anatomy, Physiology and Pharmacology, La Trobe University, Bundoora, VIC, Australia

**Keywords:** phosphate solubilization, biofertilizer, lucerne, seed microbiome, mutation, bacterial genome

## Abstract

The potential of phosphate-solubilizing microbes (PSMs) to enhance plant phosphorus uptake and reduce fertilizer dependency remains underutilized. This is partially attributable to frequent biofertilizer-farming system misalignments that reduce efficacy, and an incomplete understanding of underlying mechanisms. This study explored the seed microbiomes of nine Australian lucerne cultivars to identify and characterize high-efficiency PSMs. From a library of 223 isolates, 94 (42%) exhibited phosphate solubilization activity on Pikovskaya agar, with 15 showing high efficiency (PSI > 1.5). Genomic analysis revealed that the “high-efficiency” phosphate-solubilizing microbes belonged to four genera (*Curtobacterium*, *Pseudomonas*, *Paenibacillus*, *Pantoea*), including novel strains and species. However, key canonical genes, such as *pqq* operon and *gcd*, did not reliably predict phenotype, highlighting the limitations of *in silico* predictions. Mutagenesis of the high-efficiency isolate *Pantoea rara* Lu_Sq_004 generated mutants with enhanced and null solubilization phenotypes, revealing the potential role of “auxiliary” genes in downstream function of solubilization pathways. Inoculation studies with lucerne seedlings demonstrated a significant increase in shoot length (*p* < 0.05) following treatment with the enhanced-solubilization mutant, indicating a promising plant growth-promotion effect. These findings highlight the potential of more personalized “system-appropriate” biofertilizers and underscore the importance of integrating genomic, phenotypic, and *in planta* analyses to validate function. Further research is required to investigate links between genomic markers and functional outcomes to optimize the development of sustainable agricultural inputs.

## 1 Introduction

The agricultural industry faces a multi-pronged sustainability challenge. To meet the calorific requirements of projected population growth, the drought resilience of food systems needs to be increased while simultaneously meeting legislative and economic pressures to reduce fertilization inputs and maintain yield. Forage industry crops occupy a third of total cropland and support 4 billion livestock animals, including 1.5 billion cattle and 0.2 billion bison (Liu et al., 2023; Capstaff and Miller, 2018). In turn, the global herds of cattle and bison supply 11% of the world’s dietary protein through meat and milk (Mottet et al., 2017). However, the forage industry is vulnerable to increased drought pressure. Rising temperatures and altered rainfall patterns due to climate change have already led to widespread declines in forage persistence and productivity (Liu et al., 2023; Capstaff and Miller, 2018). This decline is particularly evident in the forage pastures of southern Australia, where the perennial ryegrass pastures that support the southern dairy region have shown decreases in water use efficiency, yield, and nitrogen content, leading to significant gaps in the feed base during dry summers (Pembleton et al., 2016; Neal et al., 2009; Liu et al., 2023; Chapman et al., 2008).

This feed gap is expected to worsen as the global climate shifts. Australian farmers can expect a 1.2-1.8°C rise in mean temperatures and a decrease in annual rainfall of 2-10% by 2050 (Hennessy et al., 2016; Pembleton et al., 2016). In preparation for the increased incidence and severity of drought these predictions herald, the south-eastern dairy region, which accounts for 80% of Australia’s milk output and is dependent on overwhelmingly rain-fed pasture systems, is investigating the incorporation of drought tolerant, supplementary forages to traditional ryegrass pastures (Pembleton et al., 2016; Cullen et al., 2012; Neal et al., 2009; Hanslow et al., 2014; Argyle, 2021). Lucerne, or alfalfa (*Medicago sativa* L.), has demonstrated increased drought tolerance, water use efficiency, and yield maintenance compared to other forages, even under simulated temperature increases of 2°C (Pembleton et al., 2016; Cullen et al., 2012; Neal et al., 2009). Colloquially known as the “queen of forage,” lucerne is a perennial leguminous forage crop well known for its high protein content, high biomass yield, excellent nutritional value, and high digestibility (Noori et al., 2018; Baudracco et al., 2011; Capstaff and Miller, 2018). Predominantly grown in arid and semi-arid regions, its popularity is also partially due to its suitability as both a standalone crop or as a part of a mixed pasture in combination with temperate and tropical grasses (Capstaff and Miller, 2018; Noori et al., 2018). In field assessments, lucerne has been demonstrated to add up to 180 kg/ha of N to the soil via its symbiotic relationship with *Ensifer meliloti* (Watson et al., 2013; Kulkarni et al., 2018). This nitrogen fixation was maintained under drought conditions and not only reduced nitrogen fertilizer requirements but also enhanced soil fertility (Capstaff and Miller, 2018). These characteristics make lucerne an ideal component of future forage systems expected to face a higher incidence of drought and is particularly well-tailored to the challenges facing rain-fed Australian dairy pastures. To support lucerne’s role in future, sustainable forage systems, research is being dedicated to maintaining yield and reducing inputs, such as fertilization (Herath Dissanayakalage et al., 2025; Tlahig et al., 2024; Niza-Costa et al., 2022; López et al., 2018; Noori et al., 2018; Noori et al., 2021; Kulkarni et al., 2018).

Fertilizer use has increased nine-fold to support the yield increases achieved in the latter half of the twenty first century (IPCC, 2019; Fiodor et al., 2021). As the nitrogen requirements of lucerne can be predominantly met by fixation, phosphorus (P), the second most ubiquitous nutritional supplement, is the limiting nutrient factor for lucerne pastures (Mei et al., 2021). The inefficiency of phosphate fertilization is well-known. Approximately two-thirds of phosphate fertilizer applied to fields undergo fixation into forms inaccessible to crops: iron or aluminum phosphate in acidic soils or calcium phosphate in alkaline soils (Mei et al., 2021; Sharma et al., 2013). In fact, estimates suggest that P accumulation in agricultural soils would be sufficient to maintain crop yields for 100 years if it were converted into plant-available forms (Sharma et al., 2013). Furthermore, excessive application of phosphate fertilizer has a variety of environmental costs, including degradation of the soil microbiome and water eutrophication, which results in algal blooms and compromised water quality (Mei et al., 2021; Sharma et al., 2013; Viscarra Rossel and Bui, 2016). To summarize, although phosphate is abundant in soil, in both inorganic and organic forms, only 1-2.5% is plant-available (Adnan et al., 2017; Viscarra Rossel and Bui, 2016). This phenomenon will only be exacerbated by the drought conditions predicted for the south Australian dairy region, as the resulting declines in soil pH further reduce quantities of plant-available phosphate (Sardans and Peñuelas, 2004; Zhang et al., 2020). Consequently, microbe-mediated solubilization of recalcitrant phosphate forms in existing soil reservoirs, in concert with nitrogen fixation by symbiotic rhizobia, will be a crucial component of strategies to reduce input costs and environmental impacts associated with forage growth (Singh et al., 2022; Amy et al., 2022).

Identifying microbes capable of mediating phosphate solubilization is not inherently challenging, as the mechanism is seemingly ubiquitously distributed in the environment. In fact, it is estimated that phosphate solubilization functionality is present in 1-50% of soil bacteria and 0.1-0.5% of soil fungi (Sharma et al., 2013). However, several challenges must still be addressed to realize the full potential of these biofertilizers in field applications. The first challenge is that despite the fact that biofertilizers have repeatedly demonstrated considerable increases in yield and nutrient mobilization, there is considerable variance in efficacy across geographies, climates, and crops (Schütz et al., 2017; da Costa et al., 2014; Ortas, 2003; Stewart and Roberts, 2012). This has led to some skepticism about their reliability (Mitter et al., 2021; Malusà et al., 2016). These inconsistencies, however, predominantly arise due to misalignment between the biofertilization microbe and the ecological context of the system it is applied to (Mitter et al., 2021; Schütz et al., 2017; Buysens et al., 2016; Ortas, 2003). Simply put, the biofertilizer industry suffers by striving to identify “one formulation for all fields” rather than choosing horses for courses (Mitter et al., 2021). As such, the application of a “personalized medicine” approach to the selection and development of microbial inoculants for unique farming systems has been suggested (Schlaeppi and Bulgarelli, 2015; Bell et al., 2019; Douds et al., 2017). Naturally, this necessitates the development of a method for the reliable identification of system-appropriate biofertilizers.

Seed-associated microbes have been demonstrated to augment the host plant’s genetic and phenotypic traits to increase nutrient acquisition, biomass accumulation, pathogenic resistance, and resilience to abiotic stress (Singh et al., 2022; Carrillo-Castañeda et al., 2002; Shade et al., 2017; Sugiyama et al., 2012; Li et al., 2020; Hone et al., 2021). The active selection and transmission of these microbial resources, both vertically and horizontally, is evidenced by the fact that seedling microbiome profiles differ significantly from the microbiome profile of the soil they are planted in, implying the seedling’s biota are inherited from the parent through the seed (van Overbeek et al., 2011; Nelson, 2018). As with other microbiomes, the members of seed microbial communities comprise core and variable components. The core microbiome, defined as a microbial community that is constantly associated with a given host genotype or a specific environment, is robust enough to remain stable across continents, crops, and selective pressures (Johnston-Monje and Raizada, 2011; Johnston-Monje et al., 2021; Berg et al., 2020). In contrast, the variable components of the microbiomes have been demonstrated to shift in response to species, genotypes, development stages, geographic locations, and environmental pressures (War et al., 2023; Shade et al., 2017; Nelson, 2018; Niza-Costa et al., 2022; Winston et al., 2014; Hone et al., 2021). This study sought to leverage the seed’s ability to curate microbial communities compatible with the host genotype and beneficial to the ecological niche of the parent plant in order to identify a “personalized,” or system-appropriate inoculant (Jochum et al., 2019; Hone et al., 2021; War et al., 2023; Marco et al., 2022). By prioritizing specificity, we aim to enhance both the consistency and efficacy of biofertilizer applications (Marco et al., 2022).

The second challenge facing biofertilizer developers is in the characterization of the mechanisms behind phosphate solubilization, or the “mode of action.” The mechanisms microorganisms employ to solubilize phosphate are variable and often cryptic. However, it is generally understood that the phosphate solubilization process falls into two major categories (Sharma et al., 2013; Alori et al., 2017). The first is the solubilization of inorganic phosphate by the production and secretion of a complex variety of organic acids (Singh et al., 2022; Pan and Cai, 2023). Which subsequently lowers the pH of the medium, enhances the chelation of P cations, competes with P for adsorption sites, and creates a soluble metal ion complex to release P (Pang et al., 2024; Qarni et al., 2021; Amy et al., 2022; Singh et al., 2022; Pan and Cai, 2023). The second is the mineralization of organic phosphorus by the secretion of enzymes, such as non-specific acid phosphatases, phytases, phosphonates, and C-P lyase, that cleave complex organic compounds to release bioavailable P (Lyagin and Efremenko, 2021; Amy et al., 2022; Singh et al., 2022; Margalef et al., 2017; Rizwanuddin et al., 2023). Other less studied methods of phosphate solubilization include H+ excretion via ammonium assimilation, extracellular oxidation via the direct oxidation pathway, chelation-driven mineral dissolution via siderophores, and an as-yet undefined microbial exopolysaccharide mechanism (Yi et al., 2008; Park et al., 2009; Song et al., 2008; Cui et al., 2022). The pathways underlying the function of these mechanisms remain poorly characterized. However, functional genomic and metagenomic studies often infer phosphate-solubilizing potential from a limited set of markers associated with acidolysis, such as *gcd* or members of the *pqq* operon, despite the incomplete picture these genes provide (Chen et al., 2024; Li et al., 2023; Wu et al., 2022a; Anzuay et al., 2024; Liang et al., 2020). Confounding this issue further are the well-known challenges associated with current genomic annotation pipelines, and functional analysis, such as taxonomy bias in standard homology methods (likely as a consequence of the overrepresentation of microbes, such as *E. coli*, in foundational *in vivo* studies) and the rapid amplification of errors that necessitate either qualification or integration with experimental data (Hamese et al., 2023; Lobb et al., 2020; Salzberg, 2019). It is clear that in the case of phosphate solubilization, the incomplete understanding of phosphate solubilization mechanisms and imperfection introduced to genomic annotation pipelines by bias and error, characterization, often *in silico* and rarely *in vivo*, has potential to be misleading (Zeng et al., 2022; Sharma et al., 2013; Alori et al., 2017).

Given southern Australia’s dairy industry’s high dependence on drought-vulnerable, fertilization-reliant forage systems, this study has three aims, (1) leverage the endemic selection processes of local commercial lucerne seed microbiomes to identify phosphate solubilizing microbes (PSMs) that could reduce the need for external inputs, (2) characterize the mode of action employed by these microbes through a combination of *in vivo* and *in silico* methods and (3) assess the candidate biofertilizer *in planta* for its material contribution to plant growth.

## 2 Materials and methods

### 2.1 Seed isolate resource

Untreated lucerne seeds from commercial cultivars were sourced from various seed companies (see Supplementary material 1). Two organically grown cultivars, Sequel and Hunter River, were sourced from Green Harvest. Organic Aurora seeds were sourced from Australian Wheatgrass, while organic Siriver was sourced from Healthforce. Ryno-6 and Force-5 cultivar seeds were sourced from AGF Seeds. Sprouts Alive cultivar seeds were sourced from Mr. Fothergills. The Trifecta cultivar seeds were obtained from Eden Seeds. Magna-959 cultivar seeds were sourced from a lucerne grower’s farm in Frances, South Australia, Australia. All seeds were stored at −20°C to preserve the viability of both seed and microbiome (Chandel et al., 2021). This study evaluated a library of 223 microbes, including 202 bacterial and 21 fungal seed isolates previously isolated from the seeds of these nine commercial cultivars (Herath Dissanayakalage et al., 2025).

### 2.2 Phosphate solubilizing activity screen

The lucerne commercial cultivar microbe library was screened for phosphate solubilizing activity using Pikovskaya agar (PVK, per liter: yeast extract, 0.5 g; dextrose, 10 g; calcium phosphate, 5 g; ammonium sulfate, 0.5 g; potassium chloride, 0.2 g; magnesium sulfate, 0.1 g; manganese sulfate, 0.0001 g; ferrous sulfate, 0.0001 g; and agar, 15 g) (Bashan et al., 2012; Gupta et al., 1994). The bacterial strains were taken from the −80°C glycerol stock, plated onto sealed R2A in four replicates, and grown for 5 days. Single bacterial colonies were taken from the isolate plates and plated onto PVK. The isolates were allowed to grow on PVK at 25°C for 10 days and then photographed to determine the phosphate solubilization index (PSI), which is equal to the diameter of the halo (mm) divided by the diameter of the colony (mm) (Berraquero et al., 1976; Marra et al., 2019).


$$P\,S\,I=\frac{c\,o\,l\,o\,n\,y\,d\,i\,a\,m\,e\,t\,e\,r+h\,a\,l\,o\,z\,o\,n\,e\,d\,i\,a\,m\,e\,t\,e\,r}{c\,o\,l\,o\,n\,y\,d\,i\,a\,m\,e\,t\,e\,r}$$

Strains with clear, “halo” zones around their colonies were identified as PSBs (phosphate solubilizing bacteria). Images were taken of each plate to allow precise digital measurements. On PVK, a PSI index of > 1.5 is considered efficient (Qarni et al., 2021; Chen and Liu, 2019; Li et al., 2019). Each isolate that demonstrated phosphate solubilization on PVK was placed in a new −80°C glycerol stock library.

### 2.3 Genomic sequencing of candidate phosphate solubilizing microbes

The genomes of candidate phosphate solubilizing microbes were sequenced. These novel strains were retrieved from −80°C glycerol storage, inoculated onto sealed R2A plates, and grown at room temperature for 5 days. A single colony was taken from each plate, grown in 30 mL nutrient broth (NB), and incubated at 25°C for 24 h at 170 cycles a minute. DNA extraction was performed using the Wizard^®^ Genomic DNA Purification Kit (A1120, Promega). The sequencing library was prepared using an in-house protocol modified from the official protocols for Oxford Nanopore Technologies (ONT, Oxford, United Kingdom) ligation-based library preparation kits (Kit 9 and Kit 14 chemistry) (Li et al., 2020). All libraries were sequenced on a MinION Mk1B platform (MIN-101B) with R9.4 and R10.4 flow cells and under the control of MinKNOW software. Following sequencing, the fast5 files that contained raw read signals were transferred to a separate, high-performance computing Linux server for base-calling and demultiplexing using ONT’s Guppy software (Version 6.2.1) with the super high accuracy model. Sequences that were of low quality (Q < 10), ONT adapters and barcodes were removed during base-calling and demultiplexing. Average nucleotide identity (ANI) was used to identify their closest genomic match and determine whether the isolate presented a novel species (ANI > 94%) or strain (Parks et al., 2021).

### 2.4 Genomic assembly and annotation

Output reads from ONT MinION were assembled using Trycycler, a high-quality, long-read-only bacterial genomic assembly tool (Wick et al., 2021). Trycycler takes advantage of the error-prone nature of long-read sequencing data by generating random subsets of total reads and using these subsets to build separate assemblies. Trycycler uses these multiple assemblies to generate a consensus sequence with far fewer sequencing errors (Wick et al., 2021). Taxonomic classification was performed using skani to assess ANI against the Genome Taxonomy Database (GTDB, Release 220) (Parks et al., 2021).

Three separate genome annotation pipelines, prokka, eggnog, and BlastKOALA, were used and manually collated to increase confidence in gene functional assignment (Seemann, 2014; Huerta-Cepas et al., 2018; Kanehisa et al., 2016). Prokka is a tool that packages external feature prediction tools such as Prodigal, RNAmmer, Aragorn, SignalP, and Infernal to annotate genomes (Seemann, 2014). Eggnog, a functional annotation tool that uses precomputed phylogenies to allow fast orthology assignments, was used to annotate the Prokka-generated protein-coding genes (Huerta-Cepas et al., 2017; Huerta-Cepas et al., 2018). Finally, BlastKOALA was used to assign gene function and reconstruct KEGG pathways using a modified BLAST algorithm to search a non-redundant dataset of pangenome sequences (Kanehisa et al., 2016). To ensure a protein was confidently assigned function, it was decided that the same KO number should appear in the BlastKOALA annotation and either the prokka or eggnog functional assignments. The fully annotated genomes were searched for key plant growth-promoting genes (PGP genes), including *gcd*, *gad*, *pqq*BCDE, *ppk*, *ppx*, *pst*SCAB, *pit*AB, *acp*A, *nap*A, *aph*A, *pho*A, *pho*D, *pho*X, *app*A, *phy*, *phn*ACDEWXYZ, and *pho*RB (Zeng et al., 2022; Pang et al., 2024; Alori et al., 2017; Sharma et al., 2013).

### 2.5 Creation of phosphate solubilization mutants

UV mutagenesis of bacterial isolates was performed using a modified version of the Barrick lab’s *E. coli* mutagenesis protocol^1^ (Deatherage et al., 2018). In a preliminary experiment, isolate Lu_Sq_004 was retrieved from −80°C glycerol storage, inoculated onto sealed R2A plates, and grown at room temperature for 5 days. A single colony was taken from the plate, grown in 30 mL nutrient broth (NB), and incubated at 25°C for 24 h at 170 cycles a minute. Using an Eppendorf tube, 1 mL of overnight culture was pelleted at 3,000 rcf for 5 min (Deatherage et al., 2018). The supernatant was removed, and the cells were resuspended in 1 mL saline solution. Two 1:10 serial dilutions were performed to reduce CFU/mL. Using thin-walled PCR tubes, 120 μL of dilute cells were exposed to a Ultra-Lum UV Crosslinker (UVAC, 230V, 50HZ, 1A) according to treatment: Control (no exposure), 60-s (exposure for 60 s), and 90-s (exposure for 90 s) (Huang et al., 2024). A series of 10 μL aliquots were plated on PVK. Control, 60-s, and 90-s treatments were also plated in serial dilutions with five replicates on sealed R2A to calculate CFU/mL and ensure that 90% cell death was maintained across rounds of mutation (where control was used as the standard) (Huang et al., 2024). The 1,030 surviving colonies were then screened for loss of phosphate solubilization activity. The colonies were plated at 25°C for 10 days and examined for the reduction or absence of clear halos. The PSI of potential increased-function, reduced-function, and null mutants were calculated according to “Phosphate solubilizing activity screen.”

### 2.6 Analyzing SNP variation between phosphate solubilization mutants

Lu_Sq_004 and its mutants were sequenced and assembled according to the “Genomic assembly and annotation” above. Following sequencing, base-calling, and variant calling were performed using Medaka (version 1.7.1), which is designed explicitly for nanopore long-read data (Lee et al., 2021). The resulting variant call format (VCF) file, containing genomic variant data for wild-type and mutant strains of Lu_Sq_004, was processed for visualization using Circos (Krzywinski et al., 2009).

### 2.7 Inoculation of Lu_Sq_004 wildtype and mutants on lucerne seedlings

Strain Lu_Sq_004 wildtype and mutants were retrieved from −80°C glycerol storage, inoculated onto sealed R2A plates, and grown at room temperature for 5 days. A single colony was taken from each plate, cultured in 30 mL nutrient broth (NB) and incubated at 25°C for 24 h at 170 cycles a minute. The following day, an optical density (OD) reading of the culture was taken and subsequently adjusted to OD_600_ = ∼ 0.5 (Herath Dissanayakalage et al., 2025; Li et al., 2020). The bacterial cells in each concentration-adjusted overnight culture were pelleted at 3,000 rcf for 5 min and resuspended in PBS. A neat solution (undiluted) and a diluted solution (phosphate-buffered saline (PBS), 10^–4^) were prepared from these resuspended cultures to create a microbial inoculant for each treatment. *Medicago sativa* cv. Siriver seeds were sterilized by soaking them in 80% ethanol for 5 min and then washing them five times in sterile distilled water and immediately placed in microbial inoculant for 4 h at 26°C in a shaking incubator (Hone et al., 2021; Herath Dissanayakalage et al., 2025). As a control, sterilized seeds were soaked in PBS under the same conditions. There were 40 seeds per treatment and 7 treatments. The seeds were then planted in individual seedling tray (5 cm x 5 cm) cells containing potting mix at a depth of 1 cm (in-house potting mix - 90.62% Biogro AGH Mix, 9.06% vermiculite, 0.12% macracote, 0.11% nitrogen, 0.08% water crystals, and 0.02 % garden lime) with one seed per cell. Seedling trays were kept in a glasshouse at 22°C with a 16-h light, 8-h dark cycle and watered lightly three times a week under seedling tray covers to maintain soil moisture. Germination rates (full seedling emergence) were calculated at 5 days after planting. After 4 weeks of growth, the cells were photographed, and the shoot lengths of the seedlings were measured. Data was statistically analyzed using a one-way ANOVA and Tukey test to detect the presence of any significant difference (*p* ≤ 0.05) between the treatments using OriginPro 2023 (Version b10.0.0.154).

## 3 Results

### 3.1 Screening lucerne seed isolates for phosphate solubilizing microbes using Pikovskaya agar

**FIGURE 1:**
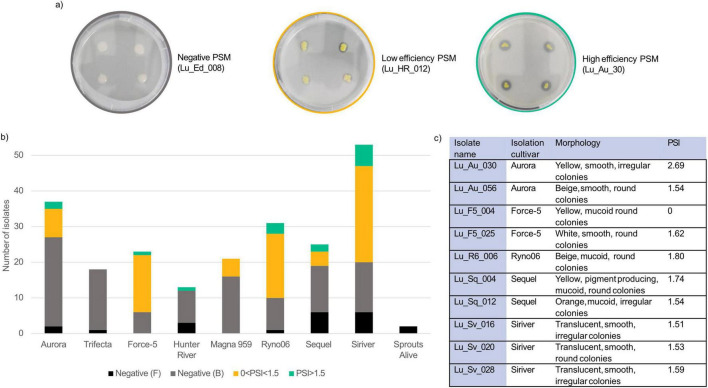
**(a)** Images of representative high-efficiency PSM (Lu_Au_030), a low-efficiency PSM (Lu_HR_012), and a non-PSM (Lu_Ed_008) after 10 days of growth, **(b)** phosphate solubilization capacity across bacterial isolates from nine commercial Australian lucerne cultivars. Isolates were categorized based on PVK screening results: negative (F – fungal) and negative (B – bacterial) indicate isolates with no zone of clearing, while 0 < PSI < 1.5 indicates isolates with low phosphate solubilization efficiency and PSI > 1.5 indicates high phosphate solubilization efficiency, respectively, based on clearing zone diameter relative to colony size, **(c)** description of colony morphologies and PSI of lucerne seed isolates chosen for sequencing.

A library of 202 bacterial and 21 fungal seed isolates was created from nine commercial Australian lucerne cultivars to identify plant-beneficial microbes (Herath Dissanayakalage et al., 2025). The 223 microbes were distributed among the lucerne cultivars as follows: Aurora (37 isolates), Trifecta (18 isolates), Force-5 (23 isolates), Sprouts Alive (2 isolates), Hunter River (13 isolates), Magna 959 (21 isolates), Ryno06 (31 isolates), Sequel (25 isolates), and Siriver (53 isolates). This collection was screened using PVK and categorized based on PSI into non-phosphate solubilizers (PSI = 0), low-efficiency phosphate solubilizers (0 < PSI < 1.5) and high-efficiency phosphate solubilizers (PSI > 1.5) (Figure 1a) to identify phosphate-solubilizing microbes (PSM). None of the fungal isolates screened produced zones of clearing of PVK. This reduced the pool of candidate phosphate biofertilisers to bacterial isolates. Overall, 94 isolates (42%) of the tested microbial isolates were positive in the PVK screen. The highest proportion of phosphate-solubilizing isolates came from the Force-5, Ryno06, and Siriver cultivars, representing 18, 23, and 35% of the total positive isolates, respectively (Figure 1b). In contrast, no phosphate solubilizers were isolated from Trifecta or Fothergill cultivars. Of the fifteen isolates with PSI > 1.5, classified as “high-efficiency solubilizers,” nine isolates that represented a range of lucerne cultivars and colony morphologies were selected for further analysis (Figure 1c). One isolate with a PSI = 0 was also sequenced as a negative control.

### 3.2 Identification and genomic characterization of phosphate solubilizing bacteria from lucerne cultivar seed isolates

To investigate mode of action in potential phosphate solubilizers *in silico*, nine high-efficiency isolates were selected, along with one negative control. The sequenced isolates belonged to four genera: *Curtobacterium*, *Pseudomonas*, *Paenibacillus*, and *Pantoea* (Table 1). The seed isolates represented two novel species and six novel strains. The *Curtobacterium* isolate was identified as a novel strain of *Curtobacterium flaccumfaciens.* The *Pseudomonas* isolates, cultured from three different cultivars, represented novel strains belonging to three separate species. All three isolates cultured from the Siriver cultivar were identified as belonging to a novel *Paenibacillus* species. A high-efficiency, pigment-producing isolate cultured from the Sequel cultivar was identified as a novel strain of *Pantoea rara*, a recently characterized species isolated from oral mouse swap (Walterson and Stavrinides, 2015). Both the negative isolate (a novel species) and one high-efficiency isolate (a novel strain) were most closely related to *Pantoea agglomerans* NBRC 102470.

**TABLE 1:** Identity and genomic characteristics of high-efficiency phosphate solubilizing lucerne seed isolates.

Genome	Identity (skani scan of GTDB)	GTDB reference	Average nucleotide identity (%)		Total length (bp)	GC content (%)	Number of genes
	*Curtobacterium flaccumfaciens* LMG 3645	GCA_013359815.1		Reference	3,827,626	70.98	3,618
Lu_Au_056			95.84	Isolate	3,714,382	71.04	3,532
	*Pseudomonas_E entomophila_A* 4A7	GCA_013523165.1		Reference	4,390,409	64.85	3,900
Lu_Au_030			98.23	Isolate	4,461,257	64.84	3,949
	*Pseudomonas_E triticicola* SWRI88	GCA_019145375.1		Reference	5,820,959	59.99	5,177
Lu_F5_025			95.02	Isolate	6,005,210	59.92	5,404
	*Pseudomonas_B oryzihabitans_E* USDA-ARS-USMARC-56511	GCA_001518815.1		Reference	4,834,356	65.11	4,460
Lu_Sq_012			98.07	Isolate	4,785,578	65.14	4,468
	*Paenibacillus amylolyticus* strain 27C64	GCA_003719335.1		Reference	6,766,424	45.69	5,944
Lu_Sv_016			87.83	Isolate	6,611,807	45.86	5,867
Lu_Sv_020			87.83	Isolate	6,611,930	45.86	5,852
Lu_Sv_028			87.83	Isolate	6,611,862	45.87	5,791
	*Pantoea rara. WMus005*	GCA_013415305.1		Reference	4,491,565	54.63	4,160
Lu_Sq_004			98.92	Isolate	4,560,210	54.72	4,355
	*Pantoea agglomerans NBRC 102470*			Reference	4,652,040	55.12	4,320
Lu_F5_004		GCA_001598475.1	90.81	Isolate	4,846,686	55.01	4,517
Lu_R6_006			97.68	Isolate	4,098,546	55.53	3,804

### 3.3 Key genes involved in inorganic phosphate solubilization and acquisition

The genomes of high-efficiency phosphate-solubilizing bacteria were annotated to identify genes involved in phosphate solubilization and acquisition (Table 2). The presence of these genes allows for the production of organic acids, releasing P immobilized by common metal cations in soil and making it bioavailable to the plant (Pan and Cai, 2023). The presence of key inorganic and organic phosphate solubilization genes varied among the high-efficiency phosphate solubilizers identified in the PVK screen of nine commercial Australian lucerne cultivars. Key genes and pathways were often missing or incomplete.

**TABLE 2:** The presence/absence of inorganic phosphate solubilization and acquisition genes of lucerne seed isolates compared to their closest GTDB genomic match.

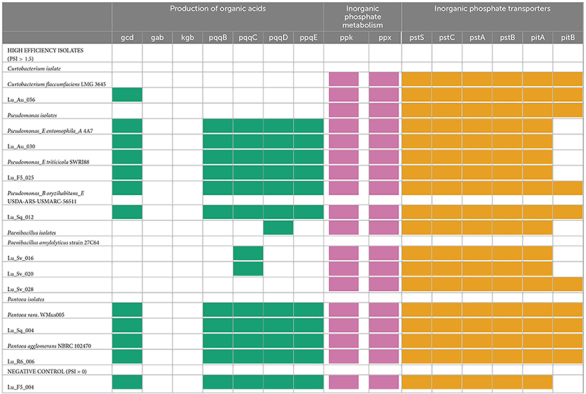

The table is divided into high-efficiency isolates and a negative control. Genes are subcategorized by function. The presence of each gene is denoted by color, while lack of color indicates gene absence.

#### 3.3.1 Production of organic acid

Pyrroloquinoline quinone (*pqq*) encodes a bacterial dehydrogenase cofactor that is essential to produce both gluconic (*gcd*) and 2-keto gluconic acid (*gad*), perhaps the most ubiquitous form of microbe-mediated acidolysis (Stites et al., 2006; Pan and Cai, 2023). These genes were identified in the genomes of all the *Pseudomonas* and *Pantoea* isolates, including the *Pantoea* strain acting as a negative genomic control (Lu_F5_004) (Table 2, “Production of organic acid”). However, the *Curtobacterium* isolate, although containing the *gcd* gene, had none of the genes needed for the pyrroloquinoline quinone co-factor and subsequent synthesis of gluconic acid. The same was true of the *Paenibacillus* isolates, where the absence of all genes save *pqq*C in Lu_Sv_016 and Lu_Sv_020, and the complete absence of all genes in Lu_Sv_028, suggests the inability to utilize gluconic acid for acidolysis (Magnusson et al., 2004). The capacity to produce 2-keto gluconic acid was not identified in any of the isolates.

#### 3.3.2 Inorganic phosphate metabolism and transport

Phosphate metabolism genes (*ppk*, *ppx*) were universally present in all isolates, supporting efficient phosphate utilization (Müller et al., 2019). However, differences in transport systems were observed. Only three of the phosphate solubilizing isolates (*C. flaccumfaciens* Lu_Au_056, *P. oryzihabitans* Lu_Sq_012, and *P. rara* Lu_Sq_004) and interestingly, the negative control, harbored all the genes needed both the high-affinity Pst and low-affinity Pit transporters (Table 2, “Inorganic phosphate transporters”). The *pit*B gene was not identified in any of the *Paenibacillus* isolates, two *Pseudomonas* isolates (Lu_Au_030, Lu_F5_025) and a *Pantoea* isolate (Lu_R6_006). The absence of *pit*B in a bacterium is hypothesized to increase the reliance of the microbe on the *pst* operon transporter and the *phn* operon, which, although more commonly associated with the movement of organic phosphate, is capable of transporting Pi during phosphate starvation (Harris et al., 2001; Stasi et al., 2019).

### 3.4 Key genes involved in organic phosphate solubilization and acquisition

The genomes were annotated to identify the presence of key genes associated with organic phosphate solubilization and acquisition (Table 3). These genes mediate enzymolysis or phosphate mineralization, the secretion of enzymes to convert unusable organic phosphate compounds into usable inorganic phosphates (Sharma et al., 2013; Margalef et al., 2017).

**TABLE 3:** The presence/absence of organic phosphate solubilization and acquisition genes of lucerne seed isolates.

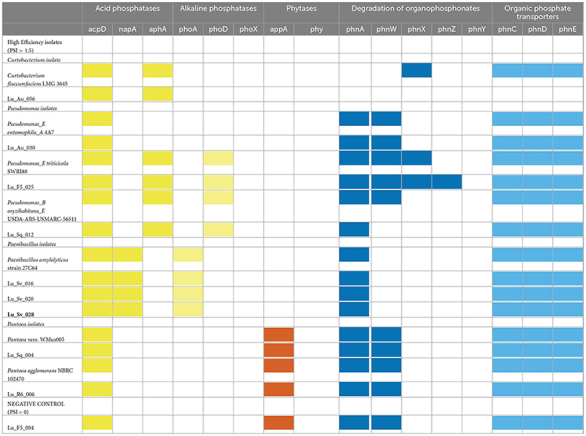

#### 3.4.1 Phosphatases

Of the three acid phosphatases selected for screening, two (*aph*A and *napA*) specialize in the phosphodiester bonds in nucleic acids, while the third (*acpD*) acts on a wider range of organic phosphate substrates (Anil and Lakshmi, 2010; Passariello et al., 2006; Thaller et al., 1995). All isolated contained *acp*D, including the negative *Pantoea* control (Table 3, “Acid phosphatase” + “Alkaline phosphatase”). The *aphA* gene was identified in the *Curtobacterium* isolate and two *Pseudomonas* isolates (Lu_F5_025, Lu_Sq_012). Only the *Paenibacillus* isolates contained the *napA* gene. Like acid phosphatases, alkaline phosphatases have varying specificity for organic phosphate substrates. PhoD is specific to the hydrolysis of phosphodiester bonds), PhoA hydrolyzes a wide range of organic phosphate compounds while phoX is hypothesized to have functions overlapping with both PhoA and PhoD (Pang et al., 2024; Sharma et al., 2013; Prabhu et al., 2019) . The *pho*A gene was exclusively identified in the genomes of the three *Paenibacillus* isolates. Two of the *Pseudomonas* isolates (Lu_F5_025, Lu_Sq_012) contained *pho*D.

#### 3.4.2 Phytases

The *appA* gene catalyzes the hydrolysis of phytate, one of the major forms of organic phosphate in soil organic matter, resulting in the release of inorganic phosphate and lower myo-inositol phosphates (Timofeeva et al., 2022; Pang et al., 2024; Singh et al., 2020). Of the two phytate genes examined in this study, only *appA* was identified in the *Pantoea* isolates, including the negative control (Table 3, “Phytase”).

#### 3.4.3 Degradation and transport of organophosphates

The *phn* operon encodes various enzymes and transporters required for phosphate utilization (Timofeeva et al., 2022). All isolates, excluding the *Curtobacterium* isolate Lu_Au_056, possessed the key ABC-type phosphonate transporter genes (*phnCDE*) (Table 3, “Degradation of organophosphonates” + “Organic phosphate transporters”) (Stasi et al., 2019). The gene *phnA*, responsible for hydrolyzing phosphoacetate into acetate and releasing inorganic phosphate, was identified in the genome of *Pseudomonas* isolate Lu_Sq_012 as well as in the *Paenibacillus* isolates (Lyagin and Efremenko, 2021). Both PhnA and PhnW, responsible for the hydrolysis of various phosphonate compounds, were identified in the *Pantoea* isolates, including the negative control and Lu_Au_030 (Lyagin and Efremenko, 2021; Zangelmi et al., 2021). Aside from *phn*Z, which was not observed in any of the high-efficiency phosphate solubilizing isolates sequenced in this study, Lu_F5_025 possessed the entire suite of *phn* genes investigated in this study.

#### 3.4.4 Regulation genes for inorganic and organic phosphate uptake

The *phoR* and *phoB* genes are key components of the two-component regulatory system that governs processes, including alkaline phosphatase expression, phosphorus uptake and transport of phosphate under low phosphorus conditions (Dai et al., 2020; Sharma et al., 2013). All *Pantoea* and *Pseudomonas* isolates, including the negative control, contained both *phoR* and *phoB*. All *Paenibacillus* isolates contained *phoR*, while *phoB* was found only in one of the three isolates (Lu_Sv_020_1).

### 3.5 Mutagenesis and characterization of phosphate solubilization in *Pantoea rara* Lu_Sq_004

**FIGURE 2:**
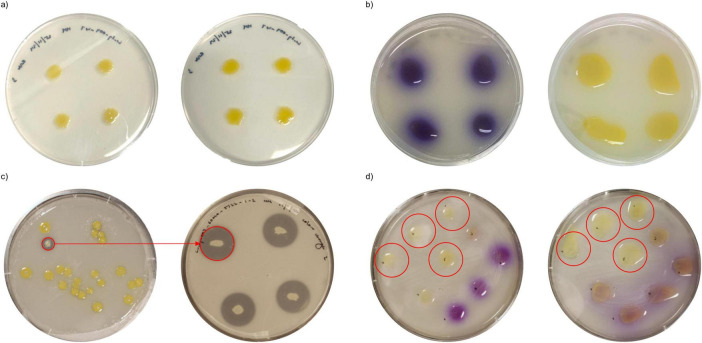
Pigment production phenotype in wildtype and mutant strains. **(a)** Lu_Sq_004_WT at day 6 (left) and day 10 (right) on R2A, **(b)** Lu_Sq_004_WT at day 6 (left) and day 10 (right) on PVK, **(c)** first identification of Lu_Sq_004_1_2 in visual screen (left) and PSI screen (right), **(d)** Lu_Sq_004 mutant pigment production screen at day 6 (left) and day 10 (right).

The combination of a high phosphate solubilization index (PSI), the presence of key inorganic solubilization/acquizition genes, and pigment production made Lu_Sq_004 the ideal candidate for a mode of action investigation through mutation. The isolate *Pantoea rara* Lu_Sq_004 produced an indigo pigment when grown on PVK, a phenomenon not observed on R2A agar (Figures 2a,b). Pigment production was visible from day 4 of growth and persisted until day 8 before disappearing by day 10. Notably, the production of this pigment was highly dependent on colony spacing, occurring only when colonies were spaced at least 18 mm apart. The pigment was not light labile.

In total, 1,030 lines were generated over two rounds of mutation. Visual assessment identified 259 lines exhibiting phenotypic variations in phosphate solubilization (as indicated by zones of clearing), pigment production, or colony morphology. Many of these mutants demonstrated reduced phosphate solubilization efficiency compared to the wild type (WT). From this screening, six mutants were selected for further investigation based on their clear phenotypes of interest. Lu_Sq_004_1_2 demonstrated a distinct colony color change from yellow in the wild type to white in the mutant (Figure 2c). Notably, Lu_Sq_004_1_2 exhibited a PSI of 4.13, significantly higher than the WT’s PSI of 1.74. Two mutants, Lu_Sq_004_4_2 and Lu_Sq_004_1_1, showed reduced phosphate solubilization efficiency relative to WT. Three mutants (Lu_Sq_004_6_3, Lu_Sq_004_6_4, and Lu_Sq_004_6_5) had negative results on PVK for phosphate solubilization and failed to produce pigment (Figure 2d).

**FIGURE 3:**
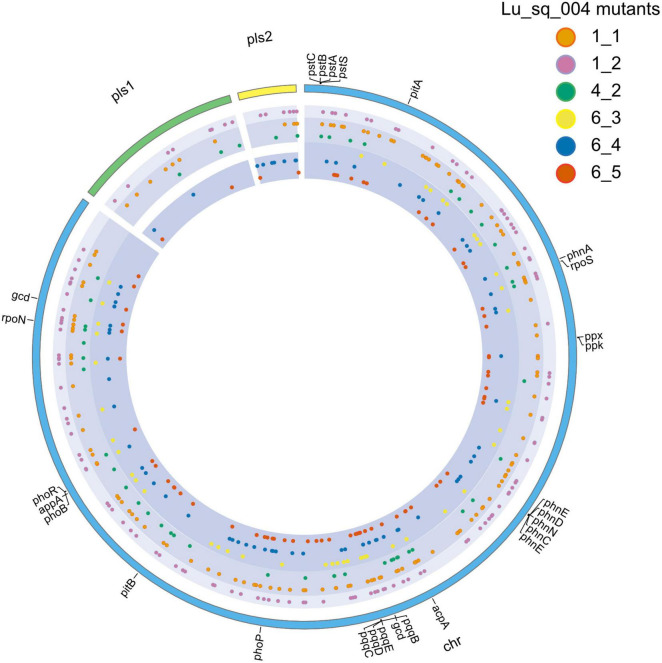
Circos plot showing the distribution of point mutations across the chromosome (chr—blue) and plasmid (pls1—green and pls2—yellow) in high-efficiency mutant Lu_Sq_004_1_2 in the outer ring, low-efficiency mutants Lu_Sq_004_1_1 and Lu_Sq_004_4_2 in the middle ring and null mutants Lu_Sq_004_6_3, Lu_Sq_004_6_4 and Lu_Sq_004_6_5 in the inner ring.

The genomes of the six selected mutants were sequenced to elucidate the genetic basis for the phenotypic variations in phosphate solubilization and pigment production. The summary characteristics of the mutant genome assemblies are described in Supplementary material 2. A total of 496 point mutations were found across the six mutation genomes. A summarized and detailed description of the placement of these point mutations, compared to the wildtype, are described in Supplementary materials 3, 4. However, none of the point mutations were within any of the key inorganic and organic phosphate solubilization genes considered in this study. In total, the three null mutants (Lu_Sq_004_6_3, Lu_Sq_004_6_4, and Lu_Sq_004_6_5) harbored 67 point mutations unique to their phenotype, including those affecting genes involved in transport, secretion, metabolism, and stress responses (Figure 3). Lu_Sq_004_6_3, which lacked both plasmids present in the wildtype, exhibited 48 point mutations, disrupting a total of 28 genes and affecting functions such as glycogen metabolism (*glgX*) and ubiquinone biosynthesis (*ubiG*). Lu_Sq_004_6_4, containing 70 mutations, demonstrated alterations in 44 genes responsible for pathways such as transport and secretion (*secY*), DNA repair (*uvrA*), and regulatory pathways (*glpR* and *rcsC*). Mutant Lu_Sq_004_6_5 had a total of 69 mutations, some located across 48 genes associated with functions such as cell wall integrity (*rfaG*), phosphate metabolism (*purH* and *sucB*), and oxidative stress response (*ydeP*). A total of 34 point mutations were identified that were unique to the low-efficiency phosphate solubilization mutants. Lu_Sq_004_1_1 contained 127 total point mutations, which affected 69 genes, some of which are involved in nutrient regulation (*putP* and *glcR*), cell wall and division (*ltaE* and *ftsW*), transport and resistance (*bmr3*) and phosphate regulation (*psuG*). Lu_Sq_004_4_2 carried 49 total mutations, affecting 28 genes, including those involved in lipid and metabolic regulation (*dgaE* and *galM*), transport and resistance (*bmr3*) and stress response (*ydfJ*). The enhanced phosphate solubilization mutant, Lu_Sq_004_1_2, carried 24 unique point mutations, including mutations within amino acid biosynthesis (*aroK* and *hisG*), DNA repair and regulation (*recX*) and drug resistance (*acrB*) genes.

### 3.6 Lucerne seed germination and shoot response to inoculation with phosphate-solubilizing mutants

**FIGURE 4:**
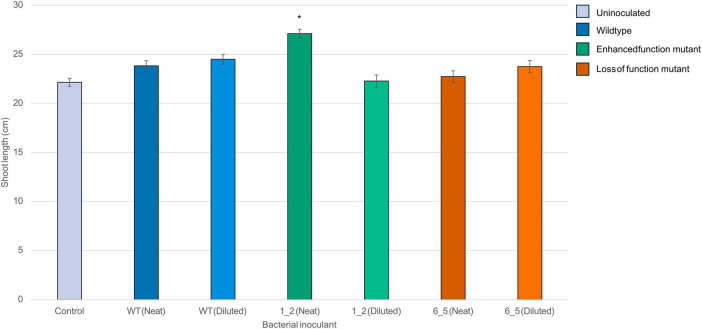
Average shoot length of 40 lucerne seedlings per treatments. Treatments were undiluted and diluted bioinoculant solutions of the wild type Lu_Sq_004_WT, Lu_Sq_004_1_2 (gain of function mutant) and Lu_Sq_004_6_5 (loss of function mutant). Bars represent the mean shoot length (cm) of seedlings treated with neat or diluted inoculants. Error bars indicate the standard error of the mean (SEM). *Represents a significant difference (*n* = 280, 7 treatments, *p* < 0.05, Tukey’s HSD test). The control group represents PBS soaked seeds, used as a baseline for comparison.

Sterilized lucerne cv. Siriver seeds were inoculated with overnight cultures of the Lu_Sq_004 wildtype, enhanced function mutant (Lu_Sq_004_1_2), and a loss of function mutant (Lu_Sq_004_6_5), and planted into an in-house potting mix. There were 40 seeds per treatment. The germination rate 5 days after planting was assessed for all conditions. Control (uninoculated) seedlings had a germination rate of 87%. The neat and diluted Lu_Sq_004_WT inoculated seedlings had germination rates of 87 and 73%. Both the neat and diluted _Lu_Sq_004_1_2 inoculated seedlings had germination rates of 87%. The neat and diluted Lu_Sq_004_6_5 had germination rates of 87 and 93%. In most conditions, the diluted treatments exhibited longer shoots than their undiluted counterparts. Seedlings treated with an undiluted overnight culture of high-efficiency phosphate solubilization mutant Lu_Sq_004_1_2 had 15.2% longer shoots than the control (Tukey’s HSD test, *p* = 0.02) (Figure 4). There were no significant differences in root growth.

## 4 Discussion

By progressing from microbial isolation to mode of action characterization to *in planta* screening, this study sought to bridge the gaps consistently found in studies designed to identify biofertilization microbes for agricultural application. A seed isolate screening highlighted a cultivar-specific selection of seed-associated phosphate solubilization bacteria in Australian commercial lucerne microbiomes. This phenomenon of host selectivity in phosphate solubilizing bacteria (PSB) curation echoes the strain-level discernment lucerne employs in rhizobial resource selection and speaks to the need for system-appropriate isolation and identification of biofertilization microbes to ensure holobiont efficacy (Wang et al., 2018; Martínez-Romero, 2009). Genomic functional analysis of PSB revealed that 32 genes in the phosphate solubilization canon failed to predict phenotype reliably in well-studied genera. The disconnect between genomic markers and phenotype underscored the limitations of relying solely on *in silico* predictions. To bridge this gap, *in vivo* functional validation of a high-efficiency PSB was undertaken using UV-mutagenesis. Enhanced and null phosphate-deficit mutants were generated, and subsequent SNP analysis identified point mutations in annotated genes that were not previously associated with phosphate solubilization. A subset of these “auxiliary” genes could be linked with the indirection regulation or downstream supply of key phosphate solubilization pathways, however, the impacts of these genes require further investigation. Finally, lucerne seedlings inoculated with the enhanced phosphate solubilization mutant had increased shoot length. Further research is required to link genomic markers to functional outcomes. Leveraging approaches like experimental evolution or mutagenesis could uncover and manipulate novel pathways, enabling the development of system-appropriate, well-characterized, high-efficacy biofertilizers.

### 4.1 Diversity and phosphate solubilization capacity of lucerne seed microbiome

The overall phosphate solubilizing capacity of Australian *Medicago sativa* seeds aligned closely with similar international studies but revealed distinct cultivar-specific differences, underscoring the role of host-driven microbial selection (Niza-Costa et al., 2022; Kirui et al., 2022; López et al., 2018). The proportion of seed isolates exhibiting phosphate solubilization in the PVK screen (42%) was consistent with similar screens conducted in lucerne on the Iberian Peninsula and Argentina (Niza-Costa et al., 2022; López et al., 2018). Similarly, the high-efficiency genera, *Curtobacterium*, *Pseudomonas*, *Paenibacillus*, and *Pantoea* highlighted in this study have been consistently identified in lucerne phosphate solubilization assays (Noori et al., 2021; López et al., 2018; Niza-Costa et al., 2022). This alignment suggests the presence of a conserved phosphate solubilization functionality in the lucerne microbiome, adapted to environmental stresses and optimized for phosphate cycling (López et al., 2018; Lemanceau et al., 2017). However, this conserved phosphate solubilization functionality varies significantly between Australian lucerne cultivars, reflecting the complexity of host-driven microbial selection (Yang et al., 2023; Winston et al., 2014; Schlemper et al., 2017; Manter et al., 2010; Mahoney et al., 2017). Between 62 and 74% of seed isolates from Force-5, Ryno06, and Siriver demonstrated phosphate solubilization activity, whereas no phosphate solubilizers were recovered from Trifecta or Sprouts Alive. These differences may reflect either the disruption of host-selection processes by breeding programs or inherent differences in the cultivars’ ability to recruit phosphate-solubilizing microbes (Gopal and Gupta, 2016; Chandel et al., 2022; Niza-Costa et al., 2022). The microbial profiles of Force-5, Ryno06, and Siriver require further investigation to elucidate the mechanisms driving phosphate-solubilizer recruitment and streamline the development of system-appropriate biofertilizers. However, among the cultivars assayed in this study, Siriver stands out, with 62% of isolates demonstrating solubilization activity and widespread industry adoption, accounting for 82% of the area planted with public varieties in Australia between 2013 and 2017 (Copping, 2020). This combination of an enriched phosphate-solubilizing microbiome and widespread cultivar usage positions Siriver as an optimal candidate for preliminary *in planta* biofertilizer screening within the Australian context.

### 4.2 *In silico* investigation of mode of action behind phosphate solubilization: insights and limitations

This study then sought to characterize the mode of action behind the high-efficiency phosphate lucerne seed isolates identified by the PVK screen using genomic functional analysis. Understanding the mode of action is crucial for ensuring efficacy and enabling system-appropriate selection of biofertilizers (Mitter et al., 2021). While functional genomics provided valuable insights into the potential mechanism of action, it also highlighted several complexities. Genes required for inorganic phosphate solubilization and acquisition through gluconic acidolysis were successfully identified in the annotated genomes of all *Pantoea* and *Pseudomonas* isolates (Hamese et al., 2023; Lobb et al., 2020; Salzberg, 2019). This is consistent with organic acid assays conducted in isolates from these genera (Mei et al., 2021). In contrast, the *Paenibacillus* and *Curtobacterium* isolates were all missing some, if not all, of the genes required for the functionality of the PQQ-GDH holoenzyme. Assuming that the inaccuracies in the functional assignment have not confounded annotation, it can, therefore, be inferred that gluconic acid production did not mediate the phosphate solubilization activity evidenced by the zone of clearing on PVK in these isolates. This highlights a gap in the familiar canon of phosphate solubilization genes surveyed in the literature, the exclusion of alternative organic acids. Gluconic and 2-keto-gluconic acid dominate genomic functional studies of microbe-mediated acidolysis (Pang et al., 2024; Qarni et al., 2021; Amy et al., 2022; Singh et al., 2022; Pan and Cai, 2023). This is despite a myriad of evidence demonstrating that multiple organic acids, including citric acid, acetic acid, lactic acid, and oxalic acid, often produced simultaneously, contribute to acidolysis (Alori et al., 2017; Wei et al., 2018; Brito et al., 2020; Marra et al., 2019). Metagenomic and *in silico* functional screens that target only gluconic acid pathways may, therefore, fail to identify high-efficiency phosphate solubilizers (Bhanja et al., 2021; Wu et al., 2022b; Chen et al., 2024). This is particularly probable in Gram-positive bacteria, such as *Paenibacillus* and *Curtobacterium*, are less likely to produce gluconic acid than their Gram-negative counterparts (Goldstein et al., 2000; Sharma et al., 2023; Park et al., 2009). Instead, in the case of the high-efficiency *Paenibacillus* and *Curtobacterium* isolates, it is likely a lesser-studied alternative acid is responsible for acidolysis (Goldstein et al., 2000; Sharma et al., 2023; Park et al., 2009). This hypothesis requires further *in vivo* investigation.

There was significant variation between the organic phosphate solubilization and acquisition gene identified in the genomes of the high-efficiency lucerne seed isolates. However, at least one phosphatase was identified in the genome of each isolate. *Pantoea* isolates Lu_Sq_004 and Lu_R6_006 contained phytase. Finally, genes encoding C-P lyases and transporters were identified in the genomes of all seed isolates, excluding *Curtobacterium* isolate Lu_Au_056. These results are consistent with experimental data that demonstrates phytase and acid phosphatase production in *Pantoea*, *Pseudomonas*, and *Paenibacillus* in low phosphate conditions (Brito et al., 2020; Mei et al., 2021). There is no experimental data defining the mechanism behind phosphate solubilization in *Curtobacterium*.

It is worth noting that not all genetic findings aligned with phenotypic observations. For instance, *P. agglomerans* Lu_F5_004, despite possessing the same key solubilization genes as the high-efficiency *P. rara* Lu_Sq_004, failed to produce a clearing zone in the PVK assay. This discrepancy underscored the limitations of relying solely on genomic annotation to predict functional outcomes (Hamese et al., 2023; Lobb et al., 2020; Salzberg, 2019). If genomic functional analysis had been used in isolation, both isolates would have been incorrectly classified as promising biofertilization candidates. This phenomenon raises important questions: Could there be misannotations of key phosphate genes, or is there a suppression of solubilization pathways in Lu_F5_004 due to unidentified factors (Hamese et al., 2023; Lobb et al., 2020; Salzberg, 2019)? It is clear that there are gaps in our current understanding of the mechanisms underlying phosphate solubilization, reinforcing the need for more fundamental research into novel gene functions and pathways to evaluate candidate organisms accurately (Marra et al., 2019; Parks et al., 2021).

### 4.3 Mutagenesis and characterization of phosphate solubilization in *Pantoea rara* Lu_Sq_004

The discrepancy in phenotype and genotype highlighted by *P. agglomerans* Lu_F5_004, a seed isolate lacking phosphate solubilization ability but containing the same number of genic markers as high-efficiency isolates, such as *P. rara* Lu_Sq_004, represents the limitations of *in silico* characterization. There are a couple of possible explanations for this phenomenon: (1) errors in the annotation itself, (2) variation within key phosphate solubilization genes impact function, or (3) significant phenotype impacts of “auxiliary” pathways not currently associated with phosphate solubilization, or (4) altered expression as a result of mutation in non-genic regulatory sites (Li et al., 2020; Hamese et al., 2023; Lobb et al., 2020; Salzberg, 2019). It is well understood that information about some phosphate solubilization mechanisms and their related genes is still limited, and some pathways are still waiting to be discovered (Amy et al., 2022; Pan and Cai, 2023). The mutation of a high-efficiency phosphate solubilizer with the aim of knocking out phosphate function without the deletion of these markers, thereby mimicking the phenotype of Lu_F5_004, could help to identify “auxiliary” genes with significant impacts on the underlying phosphate solubilization mechanism, or mode of action. The generation of this null-mutant would have the dual purpose of acting as a negative phosphate solubilization control for the validation of future *in planta* trials.

Given its close relation to *P. agglomerans* Lu_F5_004, which demonstrates that a non-solubilizing phenotype can arise without complete gene deletion, alongside its high phosphate solubilization index and pigment production, *P. rara* Lu_Sq_004 was selected as an ideal candidate for mode-of-action investigation through mutation. UV exposure yielded Lu_Sq_004 mutants that produced no zone of clearing, reduced clearing and enhanced clearing on PVK. Genomic analysis revealed that none of the phosphate solubilization mutant phenotypes resulted from mutations in the 32 key phosphate solubilization genes assessed in this study. Instead, point mutations were detected either in non-genic regions adjacent to key genes previously highlighted by the literature or in auxiliary genes that have not been directly linked to phosphate solubilization (Zeng et al., 2022). The potential phenotypic ramifications of the mutations in these auxiliary genes can be classified into four distinct categories: the establishment of a substrate bottleneck restricting gluconic acid production, the direct or indirect repression of known key phosphate pathways, a diminished capacity for acid resistance and disruption of biofilm formation.

#### 4.3.1 Phosphate solubilization-deficit mutants

Among the three identified phosphate solubilization-deficient mutants, only one, designated Lu_Sq_004_6_5, exhibited a mutation in the non-coding region between the target phosphate solubilization genes considered in this study. The point mutation lies between the *ppqB* gene and an uncharacterized gene. PqqB plays a crucial role in the functionality of the PQQ-GDH holoenzyme, therefore any alterations in its expression could significantly impact the mutant’s ability to synthesize gluconic acid (Li et al., 2014; Bhanja et al., 2021). Comparative transcriptomics may confirm whether this mutation affects *pqqB* expression. A subset of the mutated genes present across the three null mutants were identified as potential auxiliary genes that may contribute to the phosphate solubilization-deficit phenotype. The mutated *glgX* and *ubiG* genes of null-mutant Lu_Sq_004_6_3 fall into the category of genes capable of impacting gluconic acid production by damming upstream carbon. In response to Pi-limited conditions, bacteria have been demonstrated to accumulate and maintain high glycogen and ubiquinone (coenzyme Q) levels as part of adaptive metabolic shifts to capture residual Pi (Woo et al., 2010; Zhang et al., 2019). GlgX-deficient mutants have demonstrated a bottleneck in glycogen degradation, preventing the substrate flow to enzymes such as MalP and subsequent production of glucose (Dauvillée et al., 2005; Woo et al., 2010; Ma et al., 2022). Similarly, ubiG-deficient mutants proved unable to degrade CoQ10 to CoQ8 (Hsu et al., 1996). PQQ-GDH relies on ubiquinone as an electron acceptor during the oxidation of glucose to gluconic acid (Velterop et al., 1995). Without ubiquinone, PQQ-GDH may be unable to transfer electrons, leading to reduced enzymatic activity effectively (Velterop et al., 1995). The combination of substrate accumulation under Pi-limited conditions and the inability to degrade the resulting carbon reservoir could lead to a runaway sequestration effect that limits the production of gluconic acid (Ma et al., 2022; Velterop et al., 1995; Woo et al., 2010; Zhang et al., 2019).

Previous study highlights the potential of the mutated *glpR* (Lu_Sq_004_6_4), *purH* (Lu_Sq_004_6_5) and *secY* (Lu_Sq_004_6_4) genes to repress key phosphate transporters and prevent phosphatase expression, either directly or indirectly. *glpR* regulates two critical phosphate transporters, the Pst transport operon, discussed above, and the *glpQT* operon, linked to the function of alkaline phosphatases and the import of glycerol-3-phosphate across cell membranes (Lemieux et al., 2004; Martin et al., 2018; Lidbury et al., 2017; Ragot et al., 2016; Brito et al., 2020). Disruption of the *purH* gene in previous mutational studies resulted in an AICAR accumulation (Allen et al., 2002; Zeng et al., 2022). AICAR has been hypothesized to inhibit the two-component PhoBR system by preventing the autophosphorylation PhoR, subsequently affecting the expression of transporters (e.g., PstSCAB) and phosphatases (e.g., PhoD, PhoA) (Malykh et al., 2018; Zeng et al., 2022). Therefore, *glpR* and *purH* mutation could modify the expression or function of a wide range of transport systems and metabolic pathways related to phosphate solubilization. SecY, a membrane-embedded protein that facilitates the translocation of envelope proteins, is primarily known for its association with the alkaline phosphatase PhoA (Zhou et al., 2021). However, as PhoA was not identified in any *Pantoea* seed isolate genomes, a direct link between a SecY mutation and the specific phosphate solubilization of Lu_Sq_004 remains unclear (Zhou et al., 2021).

The two remaining categories of mutations that are likely responsible for the phenotypic variation observed in the three phosphate solubilization-deficient mutants include the disruption of biofilm formation associated with the *rfaG* gene (Lu_Sq_004_6_5) and the compromised acid resistance attributable to mutations in the *uvrA* (Lu_Sq_004_6_4) and *ydeP* (Lu_Sq_004_6_5) genes. The *uvrA* gene has been implicated in adaptive responses to low pH in *Streptococcus* (Hanna et al., 2001). Similarly, the deletion of *ydeP*, a putative oxidoreductase, has been linked to reduced acid resistance (Masuda and Church, 2003). With reduced acid resistance, the mutant may be unable to solubilize phosphate through acidolysis via gluconic acid. The mechanistic links behind the biofilm-mediated phosphate solubilization are not well characterized; however, the wide-ranging impact of the deep-rough phenotype *rfaG* mutants have on the biofilm formation means it is likely to disrupt this process and is worthy of further study (Pagnout et al., 2019; Sharma et al., 2013; Yi et al., 2008).

#### 4.3.2 Reduced phosphate solubilization mutants

Neither of the two low-efficiency mutants analyzed in this study had mutations within the primary phosphate solubilization genes or their adjacent non-genic regions. However, mutations in auxiliary genes in Lu_Sq_004_1_1 and Lu_Sq_004_4_2 likely contributed to reduced solubilization efficiency. Analysis of the literature indicates that the mutations most likely to be responsible are bottlenecks caused by *psuG* (Lu_Sq_004_1_1) and *dgaE* (Lu_Sq_004_4_2) and repression of key phosphate solubilization pathways by *glcR* (Lu_Sq_004_1_1). PsuG, a key enzyme in pseudouridine recycling critical for cellular nucleotide turnover (Zhou et al., 2023). In *psuG* mutants, disruptions in this pathway have been shown to influence glucose availability, potentially impacting gluconic acid levels and, subsequently, phosphate solubilization (Zhou et al., 2023; Zhang et al., 2024; Ma et al., 2022). *DgaE* operates within a mannose family phosphotransferase system and catalyzes the conversion of D-glucosaminate to 2-keto-3-deoxygluconate (Miller et al., 2013). Given that D-glucosamine can be oxidized by glucose oxidase and PQQ to form D-glucosaminate, the substrate for dgaE, a *dgaE* mutation could potentially impact downstream gluconic acid production (Miller et al., 2013). Mutations in *glcR*, a transcriptional regulator of glucose metabolism, reduce organic acid production by disrupting the glcR-phoC operon response, indirectly diminishing solubilization potential by preventing the upregulation of phosphate solubilization pathways (Morabbi Heravi et al., 2019; Ma et al., 2022; Leyn et al., 2013).

#### 4.3.3 Enhanced phosphate solubilization mutants

Our experimentation revealed an enhanced phosphate-solubilizing effect following mutagenesis. UV-induced mutation in Lu_Sq_004_1_2 resulted in a 2.4-fold increase in the phosphate solubilization index. None of the mutations occurred in the non-genic regions adjacent to the key solubilization genes focused on in this study. However, a point mutation was identified in the *hisG*, which has the potential to repress key phosphate solubilization pathways indirectly. The *hisG* gene produces an ATP-PR transferase that catalyzes the formation of phosphoribosyl-ATP from phosphoribosyl pyrophosphate and ATP in the first step of the histidine biosynthesis pathway (Malykh et al., 2018). Histidine biosynthesis is accompanied by an equimolar AICAR generation (Malykh et al., 2018). As previously discussed, AICAR accumulation inhibits the two-component PhoBR system, indirectly regulating phosphate transport systems and phosphatases (Malykh et al., 2018). Furthermore, histidine accumulation has been observed in *pitA*-mutations, suggesting a link between histidine biosynthesis and phosphate solubilization that goes beyond PhoRB regulation. These findings suggest that UV-induced mutations in genes like *hisG*, which may indirectly affect phosphate solubilization via histidine biosynthesis and AICAR accumulation, could enhance the solubilization phenotype (Malykh et al., 2018).

Comprehensive transcriptomic analysis should be conducted to evaluate these predictions and investigate the potential of regulation changes caused by mutations in non-genic regions that could not be analyzed using genomic data. Ultimately, this study highlights the complexity of accurately predicting gene function from the genome alone and indicates that while auxiliary genes may not have direct roles in solubilization, their downstream contributions to the phosphate solubilization phenotype could be more significant than previously understood.

### 4.4 *In planta* assessment of *Pantoea rara* Lu_Sq_004 mutants: contributions to lucerne growth

The complexity of phosphate solubilization means it is crucial that once a potential PSM is identified, it is specifically tested to determine its mode of action and material contribution to plant phosphorus nutrition, as not all phosphate solubilization mechanisms translate directly into a plant growth promotion effect (Sharma et al., 2013; Bashan et al., 2012; Elhaissoufi et al., 2020). While mutations that enhance phosphate solubilization show promise, the primary aim remains to identify a candidate biofertilizer, making it essential to confirm that increased solubilization translates to enhanced phosphate availability in lucerne. While field studies offer the most conclusive insights for *in planta* assessments, a glasshouse study was selected for initial evaluation of plant responses under controlled conditions. Inoculation with Lu_Sq_004_WT, despite its status as a high-efficiency phosphate solubilizer, did not translate to increased growth in treated seedlings, highlighting the need for *in planta* validation of PSM candidates. However, significant differences in shoot length were observed in 4-week-old seedlings inoculated with the high-efficiency mutant Lu_Sq_004_1_2, compared to both the wild type and the phosphate solubilization-deficient mutant Lu_Sq_004_6_5. These findings align with previous studies showing a significant increase (*p* < 0.05) in shoot length among PSB-bioprimed seeds (Musa and Ikhajiagbe, 2023; Mei et al., 2021). While it cannot be said definitively that the increase in shoot length is directly attributable to phosphate solubilization, as phosphate solubilizers are often capable of other plant growth promotion effects, the enhanced growth observed in the gain-of-function mutant compared to both the control and the null-phosphate phenotype is a promising indication (Sharma et al., 2013; Musa and Ikhajiagbe, 2023; Mei et al., 2021).

Members of the *Pantoea* genera have consistently been identified as core components of not only of geographically disparate lucerne seed microbiomes, but also related legumes (Noori et al., 2021; López et al., 2018; Niza-Costa et al., 2022; Chandel et al., 2022; Sulesky-Grieb et al., 2024). Furthermore, as *in planta* testing was conducted using a non-sterile potting mix, there is evidence of functional persistence amidst soil microbiome competition (Ortas, 2003; Biró et al., 2000). The persistence of functional traits in the phosphate solubilization mutants, even amidst competitive pressures from the native soil microbiome, demonstrates their potential for reliable integration into agricultural systems, where similar ecological dynamics are unavoidable (Ortas, 2003; Biró et al., 2000). While this seeming stability requires further field testing, this indicates the robustness the candidate biofertilizer and the potential for applications in complex, real-world agricultural settings.

The study lays out a method that can be applied to a diverse range of target crops and agroecological systems. By isolating from locally grown seeds of the target crop, the endemic microbial resources naturally curated by the host plant can be leveraged to create a pool of system-appropriate candidates. *In vivo* experimentation can further the understanding gained from *in silico* predictions, and, in the case of mutational work, generate mutants that allow for more precise field trial controls. It is worth considering that isolating such system-appropriate microbes from seed microbiomes relies on preserving host-microbe relationships and selection strategies. Lucerne, for example, has not been subject to rigorous domestication, unlike other leguminous species, such as lentil and soybean (Guerra-Garcia et al., 2022; Niza-Costa et al., 2022). As a result, the relationship between beneficial microbiomes and the host crop is remarkably well-preserved (Niza-Costa et al., 2022). The microbiome of close and crop-wild relatives have been highlighted as the potential “next best” pool of agroecological candidates in cases where the target crop’s host-microbe relationships have been degraded by breeding (Chen et al., 2021; Compant et al., 2025). It is possible that seed-associated microbes of commercial Australian lucerne cultivars while tailored to the host, may provide a pool of system-appropriate phosphate solubilizers suitable for the Australian agroecological context and therefore, broader application in legumes where the endemic microbial resources and holobiont relationships have been eroded (Niza-Costa et al., 2022; Kirui et al., 2022; El Moukhtari et al., 2022; Carrillo-Castañeda et al., 2002; Liu et al., 2023; Marco et al., 2022).

## 5 Conclusion

This study demonstrates the critical importance of system-appropriate isolation, genomic functional analysis, and *in planta* testing in bridging the gaps in biofertilizer development for agricultural application. By exploring the phosphate-solubilizing capacity of an Australian commercial lucerne seed isolate library, it became evident that host-driven microbial selection plays a pivotal role in shaping cultivar-specific microbiomes. Three cultivars, Force-5, Ryno06, and Siriver emerged as promising candidates for biofertilizer development due to their enriched phosphate-solubilizing communities. While genomic functional analysis provided valuable insights into the phosphate solubilization mechanisms utilized by some seed isolates belonging to some genera (such as *Pantoea* and *Pseudomonas*), it also revealed the limitations of relying solely on canonical markers such as PQQ-associated gluconic acid pathways in elucidating the mechanisms used in others. Mutagenesis highlighted potential “auxiliary” genes that suggest a complex genetic landscape with significant impacts on overall function. These genes and their associated pathways require further transcriptomic and functional experimentation to elucidate their contribution to phosphate solubilization. Furthermore, seeds inoculated with an enhanced-efficiency mutant generated from high-efficiency isolate *P. rara* Lu_Sq_004 demonstrated increased shoot growth, compared to null mutant inoculated and uninoculated control seeds in *in planta* greenhouse trials. Ultimately, this study emphasizes the importance of integrating genomic, functional, and ecological perspectives to identify system-appropriate biofertilizers and enhance agricultural sustainability. Leveraging advanced approaches like experimental evolution and transcriptomics, alongside rigorous field trials, will be critical for realizing the full potential of phosphate-solubilizing microbes in diverse agricultural contexts.

## Data Availability

The datasets presented in this study can be found in online repositories. The names of the repository/repositories and accession number(s) can be found at: https://www.ncbi.nlm.nih.gov/, PRJNA1210666.

## Footnotes

1
https://barricklab.org/twiki/bin/view/Lab/ProtocolsUVLibrary
