# Supplementary material for: Often in silico, rarely in vivo: characterizing endemic plant-associated microbes for system-appropriate biofertilizers
Source: Front Microbiol. 2025 Apr 28;16:1568162. doi: 10.3389/fmicb.2025.1568162 (PMC12066602; doi:10.3389/fmicb.2025.1568162)
Supplement: Supplementary file 1 [file Table_1.docx]

Often in silico, rarely in vivo: characterizing endemic plant-associated microbes for system-appropriate biofertilizers - Supplementary Information

Supplementary 1: Lucerne commercial cultivars

| Source | Lucerne cultivar | Features |
| --- | --- | --- |
| Green Harvest | Sequel | Organically grown, untreated |
| Green Harvest | Hunter River | Organically grown, untreated |
| Eden Seeds | Trifecta | Organically grown |
| Australian Wheatgrass | Aurora | Certified organic |
| Healthforce | Siriver | Organically grown, untreated |
| AGF Seeds | Ryno 6 | Untreated/uninoculated |
| AGF Seeds | Force 5 | Untreated/uninoculated |
| Sprouts Alive | Mr Fothergills | Untreated/uninoculated |
| A farm in Frances, SA | Magna 959 | Untreated |

Supplementary 2: Genomic characteristics of Pnr_Lu_Sq_004

|  | No. of mutations | Phenotype | Chromosome | Plasmids | Genome size | Protein coding genes |
| --- | --- | --- | --- | --- | --- | --- |
| Pnr_Lu_Sq_004_WT | - | - | 3,7143,82 | 1: 472,234; 2: 159,929 | 4,560,210 | 4255 |
| PnrLu_Sq_004_1_2 | 133 | Colour change, high efficiency | 3,928,134 | 1: 472,239, 2:159,932 | 4,560,305 | 4190 |
| PnrLu_Sq_004_1_1 | 127 | Low efficiency | 3,928,116 | 1: 472,243, 2: 159,933 | 4,560,292 | 4183 |
| PnrLu_Sq_004_4_2 | 49 | Low efficiency | 3,927,986 | 1: 472,231, 2:159,932 | 4,560,140 | 4215 |
| PnrLu_Sq_004_6_3 | 48 | Negative | 3,928,008 | N/A | 3,928,008 | 3645 |
| PnrLu_Sq_004_6_4 | 70 | Negative | 3,928,027 | 1: 472,230, 2: 159,921 | 4,560,178 | 4237 |
| PnrLu_Sq_004_6_5 | 69 | Negative | 3,928,010 | 1: 472,233, 2: 159,927 | 4,560,170 | 4246 |

Supplementary 3: Summary of genes containing SNP variations between Lu_Sq_004_WT and its mutants

| Phenotype | Name | Total mutations | Unique mutations | Intra-gene mutation | Inter-gene mutation | No of genes affected | Genes |
| --- | --- | --- | --- | --- | --- | --- | --- |
| Negative | Lu_Sq_004_6_3 | 48 | 67 | 45 | 3 | 28 | aldA, betA, cntO_1, eamA_2, garD_2, glgX_2, glnQ_1, gltP, greA, lpdA, nsrR, paoD, pfkA_2, ppsA, prop_4, purH, rsxG, sasA_3, secY, sfmC_1, ssuE, tamA, ubiG, yafV, ybaY, ydeP_2, yfdH, yhcB |
|  | Lu_Sq_004_6_4 | 70 |  | 62 | 8 | 42 | amiA, asnB, bioD1_1, clsC, cobB, davT_1, dctP, emrB_1, fabD_2, fliG, garD_2, glnQ_1, glpR_1, gltP, gshA, guaD_2, kdsA, lpdA, lpxP, mgsA, mlaC, mntB_4, oppA_1, PGdx, phoQ, potA_1, proP_4, purH, puuA, rcsC_1, rsxG, rutF, scrB, secY, tamA, trmL_2, ttcA, uvrA_2, yajL_2, ybaY, ydeP_2, yhcB |
|  | Lu_Sq_004_6_5 | 69 |  | 59 | 10 | 47 | amiA, asnB, bcr_2, btuR, cobB, crtY, cysW, dapA_2, dcuA, dut, eamA_2, fabD_2, flgK, galP_3, gltP, lgoD, lpxP, matP, mfd, motA, mreB, norW, oppA_1, oqxB26, patB, PGdx, pldB, potA_1, proP_4, purA, purH, rcsC_1, rfaG, rhaT, rpiA_1, rseA, sapA, sohB, sucB, ubiG, yajL_2, ybaY, ydeP_2, ydiV, yecS_2, yfcF, yohC |
| Low-efficiency | Lu_Sq_004_1_1 | 127 | 34 | 69 | 58 | 68 | acrF, aldA, argP_2, bioD1_1, clpA, cntO_1, cobB, cra_2, damX, davT_1, dctM, dctP, dgt, dinF_2, dmlR_8, dnaX, dsbD_2, dusA, dut, eamA_2, ftsW, galP_3, garD_2, glcR_1, glnQ_1, gltP, greA, gshA, gsiD_3, gstB_2, hemN, hmp, lpdA, ltaE, metQ_2, mglA_4, mnmC_2, mobB, motA, mreB, msrC, mutY, narG_1, paoD, patB, pbpG_2, PGdx, pldB, potA_1, proP_4, psuG_1, ptsG, purA, purH, putP, pxpA, rhtC_3, rsxG, rutF, sfmC_1, tamA, yafV, yajL_2, ybaK, ycaD_1, yfcF, yfdH, yhcB |
|  | Lu_Sq_004_4_2 | 49 |  | 40 | 9 | 28 | aldA, bioD1_1, bmr3, cobB, dacC, dbpA, dctM, dgaE, dorA, galM, galP_3, gltP, gshA, gstB_2, mlaC, narG_1, nsrR, patB, pbpG_2, potA_1, purH, puuA, rhaT, rhtC_3, secF, ybaY, ydfJ, yfdH |
| Enhanced | Lu_Sq_004_1_2 | 133 | 24 | 106 | 27 | 71 | acrB_2, acrF, amiA, argP_2, aroK, bfr, bioD1_1, bmr3, clpA, cntO_1, cpdB, cpxR_2, cra_2, cysM, damX, davT_1, dctM, dcuA, dgt, dinF_2, dnaX, dsbD_2, dut, eamA_2, fabD_2, flgK, garD_2, greA, gshA, hemN, hflX, hisG, hmp, htpX_1, lgoD, lpdA, lpxP, mglA_4, mlaC, mnmC_2, motA, mreB, msrC, mutY, narG_1, nsrR, paoD, patB, pbpG_2, PGdx, pldB, proP_4, ptsG, purA, puuA, pxpA, recX, rhaT, rluB, rutF, ssuE, tamA, tsgA, ttcA, yafV, ybaK, ycaD_1, yfcF, yfdH, yhcB, yigB |

Supplementary 4: Medaka SNP variations between Lu_Sq_004_WT and its mutants

| Location | Nucleotide | Mutant | Cat | Within/Between | Gene ID | Gene name |
| --- | --- | --- | --- | --- | --- | --- |
| plasmid2 | 4214 | 6_3 | Neg | Within | GMKMPLAH_04139 | ydeP_2 |
| plasmid2 | 4214 | 6_4 | Neg | Within | GMKMPLAH_04139 | ydeP_2 |
| plasmid2 | 4214 | 6_5 | Neg | Within | GMKMPLAH_04139 | ydeP_2 |
| plasmid2 | 27052 | 6_4 | Neg | Within | GMKMPLAH_04158 | ydeP_2 |
| plasmid2 | 50212 | 1_1 | Low | Between | GMKMPLAH_04178, GMKMPLAH_04179 | hns_4, unknown |
| plasmid2 | 60661 | 6_4 | Neg | Within | GMKMPLAH_04188 | guaD_2 |
| plasmid2 | 65981 | 1_1 | Low | Within | GMKMPLAH_04193 | unknown |
| plasmid2 | 68951 | 6_4 | Neg | Between | GMKMPLAH_04194, GMKMPLAH_04195 | unknown, unknown |
| plasmid2 | 87750 | 4_2 | Low | Within | GMKMPLAH_04210 | unknown |
| plasmid2 | 107090 | 6_4 | Neg | Within | GMKMPLAH_04228 | uvrA_2 |
| plasmid2 | 115677 | 6_3 | Neg | Within | GMKMPLAH_04237 | sasA_3 |
| plasmid2 | 120374 | 1_1 | Low | Within | GMKMPLAH_04239 | acrF |
| plasmid2 | 120374 | 1_2 | High | Within | GMKMPLAH_04239 | acrF |
| plasmid2 | 138393 | 1_1 | Low | Within | GMKMPLAH_04255 | unknown |
| plasmid2 | 148915 | 6_4 | Neg | Within | GMKMPLAH_04264 | unknown |
| plasmid2 | 149896 | 1_2 | High | Within | GMKMPLAH_04265 | cra_2 |
| plasmid2 | 149896 | 1_1 | Low | Within | GMKMPLAH_04265 | cra_2 |
| plasmid2 | 151270 | 6_4 | Neg | Within | GMKMPLAH_04266 | scrB |
| plasmid2 | 159363 | 1_2 | High | Between | GMKMPLAH_04272 | unknown |
| plasmid2 | 159363 | 1_1 | Low | Between | GMKMPLAH_04272 | unknown |
| plasmid2 | 159363 | 6_5 | Neg | Between | GMKMPLAH_04272 | unknown |
| plasmid2 | 159363 | 4_2 | Low | Between | GMKMPLAH_04272 | unknown |
| plasmid1 | 27862 | 1_1 | Low | Within | GMKMPLAH_03706 | unknown |
| plasmid1 | 29651 | 6_5 | Neg | Within | GMKMPLAH_03710 | unknown |
| plasmid1 | 37151 | 6_4 | Neg | Within | GMKMPLAH_03720 | mntB_4 |
| plasmid1 | 41254 | 1_2 | High | Within | GMKMPLAH_03725 | unknown |
| plasmid1 | 87315 | 1_2 | High | Within | GMKMPLAH_03769 | paoD |
| plasmid1 | 87315 | 1_1 | Low | Within | GMKMPLAH_03769 | paoD |
| plasmid1 | 87315 | 6_3 | Neg | Within | GMKMPLAH_03769 | paoD |
| plasmid1 | 145991 | 1_2 | High | Within | GMKMPLAH_03823 | unknown |
| plasmid1 | 208401 | 1_2 | High | Within | GMKMPLAH_03887 | unknown |
| plasmid1 | 235436 | 6_4 | Neg | Within | GMKMPLAH_03912 | unknown |
| plasmid1 | 235436 | 1_2 | High | Within | GMKMPLAH_03912 | unknown |
| plasmid1 | 235436 | 4_2 | Low | Within | GMKMPLAH_03912 | unknown |
| plasmid1 | 235436 | 6_3 | Neg | Within | GMKMPLAH_03912 | unknown |
| plasmid1 | 243804 | 1_1 | Low | Within | GMKMPLAH_03921 | metQ_2 |
| plasmid1 | 261210 | 1_2 | High | Within | GMKMPLAH_03936 | dsbD_2 |
| plasmid1 | 261210 | 1_1 | Low | Within | GMKMPLAH_03936 | dsbD_2 |
| plasmid1 | 379928 | 6_5 | Neg | Within | GMKMPLAH_04042 | crtY |
| plasmid1 | 387675 | 1_1 | Low | Within | GMKMPLAH_04052 | mnmC_2 |
| plasmid1 | 387675 | 1_2 | High | Within | GMKMPLAH_04052 | mnmC_2 |
| plasmid1 | 393056 | 1_1 | Low | Within | GMKMPLAH_04058 | rhtC_3 |
| plasmid1 | 393056 | 4_2 | Low | Within | GMKMPLAH_04058 | rhtC_3 |
| plasmid1 | 434568 | 1_1 | Low | Within | GMKMPLAH_04101 | psuG_1 |
| plasmid1 | 458179 | 1_1 | Low | Within | GMKMPLAH_04120 | gstB_2 |
| plasmid1 | 458179 | 4_2 | Low | Within | GMKMPLAH_04120 | gstB_2 |
| chr1 | 55814 | 1_1 | Low | Between | GMKMPLAH_00053, GMKMPLAH_00054 | atpI, rsmG |
| chr1 | 55814 | 1_2 | High | Between | GMKMPLAH_00053, GMKMPLAH_00054 | atpI, rsmG |
| chr1 | 55814 | 4_2 | Low | Between | GMKMPLAH_00053, GMKMPLAH_00054 | atpI, rsmG |
| chr1 | 83253 | 6_4 | Neg | Between | GMKMPLAH_00076, GMKMPLAH_00077 | unknown, unknown |
| chr1 | 83253 | 1_1 | Low | Between | GMKMPLAH_00076, GMKMPLAH_00077 | unknown, unknown |
| chr1 | 83253 | 1_2 | High | Between | GMKMPLAH_00076, GMKMPLAH_00077 | unknown, unknown |
| chr1 | 83253 | 4_2 | Low | Between | GMKMPLAH_00076, GMKMPLAH_00077 | unknown, unknown |
| chr1 | 83786 | 1_1 | Low | Within | GMKMPLAH_00077 | mobB |
| chr1 | 92748 | 1_1 | Low | Within | GMKMPLAH_00085 | hemN |
| chr1 | 92748 | 1_2 | High | Within | GMKMPLAH_00085 | hemN |
| chr1 | 118549 | 1_2 | High | Within | GMKMPLAH_00106 | lpxP |
| chr1 | 118549 | 6_5 | Neg | Within | GMKMPLAH_00106 | lpxP |
| chr1 | 118549 | 6_4 | Neg | Within | GMKMPLAH_00106 | lpxP |
| chr1 | 124855 | 6_5 | Neg | Within | GMKMPLAH_00113 | dut |
| chr1 | 124855 | 1_1 | Low | Within | GMKMPLAH_00113 | dut |
| chr1 | 124855 | 1_2 | High | Within | GMKMPLAH_00113 | dut |
| chr1 | 131746 | 6_5 | Neg | Within | GMKMPLAH_00122 | rfaG |
| chr1 | 153903 | 4_2 | Low | Between | GMKMPLAH_00145 | unknown |
| chr1 | 189171 | 1_2 | High | Within | GMKMPLAH_00176 | PGdx |
| chr1 | 189171 | 6_5 | Neg | Within | GMKMPLAH_00176 | PGdx |
| chr1 | 189171 | 1_1 | Low | Within | GMKMPLAH_00176 | PGdx |
| chr1 | 189171 | 6_4 | Neg | Within | GMKMPLAH_00176 | PGdx |
| chr1 | 202966 | 6_3 | Neg | Within | GMKMPLAH_00188 | unknown |
| chr1 | 202966 | 1_1 | Low | Within | GMKMPLAH_00188 | unknown |
| chr1 | 202966 | 1_2 | High | Within | GMKMPLAH_00188 | unknown |
| chr1 | 202966 | 4_2 | Low | Within | GMKMPLAH_00188 | unknown |
| chr1 | 240556 | 1_2 | Low | Within | GMKMPLAH_00222 | lgoD |
| chr1 | 240556 | 6_5 | Neg | Within | GMKMPLAH_00222 | lgoD |
| chr1 | 253806 | 6_5 | Neg | Between | GMKMPLAH_00234, GMKMPLAH_00235 |  |
| uuchr1 | 280795 | 1_1 | Low | Between | GMKMPLAH_00257, GMKMPLAH_00258 | rlmJ, prlC |
| chr1 | 280795 | 1_2 | High | Between | GMKMPLAH_00257, GMKMPLAH_00258 | rlmJ, prlC |
| chr1 | 289592 | 1_2 | High | Within | GMKMPLAH_00266 | unknown |
| chr1 | 289592 | 1_1 | Low | Within | GMKMPLAH_00266 | unknown |
| chr1 | 289592 | 6_3 | Neg | Within | GMKMPLAH_00266 | unknown |
| chr1 | 373476 | 6_4 | Neg | Within | GMKMPLAH_00342 | glpR_1 |
| chr1 | 391572 | 1_2 | High | Between | GMKMPLAH_00357, GMKMPLAH_00358 | hslR, yrfG |
| chr1 | 402859 | 1_2 | High | Within | GMKMPLAH_00369 | aroK |
| chr1 | 404257 | 1_2 | High | Within | GMKMPLAH_00370 | damX |
| chr1 | 404257 | 1_1 | Low | Withih | GMKMPLAH_00370 | damX |
| chr1 | 414257 | 1_2 | High | Within | GMKMPLAH_00379 | tsgA |
| chr1 | 443530 | 1_2 | High | Within | GMKMPLAH_00413 | bfr |
| chr1 | 450550 | 1_2 | High | Within | GMKMPLAH_00426 | unknown |
| chr1 | 457706 | 6_4 | Neg | Within | GMKMPLAH_00437 | secY |
| chr1 | 457706 | 6_3 | Neg | Within | GMKMPLAH_00437 | secY |
| chr1 | 477221 | 1_1 | High | Within | GMKMPLAH_00465 | glnQ_1 |
| chr1 | 477221 | 6_3 | Neg | Within | GMKMPLAH_00465 | glnQ_1 |
| chr1 | 477221 | 6_4 | Neg | Within | GMKMPLAH_00465 | glnQ_1 |
| chr1 | 492760 | 1_2 | High | Within | GMKMPLAH_00480 | mreB |
| chr1 | 492760 | 6_5 | Neg | Within | GMKMPLAH_00480 | mreB |
| chr1 | 492760 | 1_1 | Low | Within | GMKMPLAH_00480 | mreB |
| chr1 | 527542 | 1_2 | High | Within | GMKMPLAH_00512 | hflX |
| chr1 | 531659 | 1_2 | High | Within | GMKMPLAH_00516 | purA |
| chr1 | 531659 | 6_5 | Neg | Within | GMKMPLAH_00516 | purA |
| chr1 | 531659 | 1_1 | Low | Within | GMKMPLAH_00516 | purA |
| chr1 | 533431 | 1_2 | High | Within | GMKMPLAH_00517 | nsrR |
| chr1 | 533431 | 6_3 | Neg | Within | GMKMPLAH_00517 | nsrR |
| chr1 | 533431 | 4_2 | Low | Within | GMKMPLAH_00517 | nsrR |
| chr1 | 547330 | 1_2 | High | Within | GMKMPLAH_00530 | cpdB |
| chr1 | 552626 | 1_1 | Low | Within | GMKMPLAH_00535 | tamA |
| chr1 | 552626 | 6_4 | Neg | Within | GMKMPLAH_00535 | tamA |
| chr1 | 552626 | 1_2 | High | Within | GMKMPLAH_00535 | tamA |
| chr1 | 552626 | 6_3 | Neg | Within | GMKMPLAH_00535 | tamA |
| chr1 | 566759 | 6_5 | Neg | Between | GMKMPLAH_00548,GMKMPLAH_00549 | cybC, unknown |
| chr1 | 571495 | 4_2 | Low | Within | GMKMPLAH_00555 | dgaE |
| chr1 | 628692 | 4_2 | Low | Between | GMKMPLAH_00618, GMKMPLAH_00619 | unknown |
| chr1 | 634734 | 1_2 | High | Within | GMKMPLAH_00626 | unknown |
| chr1 | 684995 | 1_1 | Low | Within | GMKMPLAH_00675 | dctM |
| chr1 | 684995 | 4_2 | Low | Within | GMKMPLAH_00675 | dctM |
| chr1 | 684995 | 1_2 | High | Within | GMKMPLAH_00675 | dctM |
| chr1 | 687563 | 1_1 | Low | Within | GMKMPLAH_00677 | dctP |
| chr1 | 687563 | 6_4 | Neg | Within | GMKMPLAH_00677 | dctP |
| chr1 | 699496 | 1_1 | Low | Within | GMKMPLAH_00687 | unknown |
| chr1 | 699496 | 6_5 | Neg | Within | GMKMPLAH_00687 | unknown |
| chr1 | 699496 | 6_3 | Neg | Within | GMKMPLAH_00687 | unknown |
| chr1 | 715517 | 1_1 | Low | Within | GMKMPLAH_00698, GMKMPLAH_00699 | scmP_1, prcF |
| chr1 | 715517 | 6_4 | Neg | Within | GMKMPLAH_00698, GMKMPLAH_00699 | scmP_1, prcF |
| chr1 | 715517 | 1_2 | High | Within | GMKMPLAH_00698, GMKMPLAH_00699 | scmP_1, prcF |
| chr1 | 715517 | 6_3 | Neg | Within | GMKMPLAH_00698, GMKMPLAH_00699 | scmP_1, prcF |
| chr1 | 737282 | 1_1 | Low | Within | GMKMPLAH_00718 | unknown |
| chr1 | 737282 | 1_2 | High | Within | GMKMPLAH_00718 | unknown |
| chr1 | 737282 | 6_4 | Neg | Within | GMKMPLAH_00718 | unknown |
| chr1 | 737283 | 6_3 | Neg | Within | GMKMPLAH_00718 | unknown |
| chr1 | 750202 | 1_1 | Low | Within | GMKMPLAH_00731 | mutY |
| chr1 | 750202 | 1_2 | High | Within | GMKMPLAH_00731 | mutY |
| chr1 | 764935 | 1_1 | Low | Within | GMKMPLAH_00748 | galP_3 |
| chr1 | 764935 | 6_5 | Neg | Within | GMKMPLAH_00748 | galP_3 |
| chr1 | 764935 | 4_2 | High | Within | GMKMPLAH_00748 | galP_3 |
| chr1 | 782582 | 6_5 | Neg | Within | GMKMPLAH_00762 | rpiA_1 |
| chr1 | 820049 | 1_1 | Low | Within | GMKMPLAH_00799, GMKMPLAH_00800 | mutH, rppH |
| chr1 | 820049 | 1_2 | High | Within | GMKMPLAH_00799, GMKMPLAH_00800 | mutH, rppH |
| chr1 | 851748 | 1_2 | High | Between | GMKMPLAH_00827, GMKMPLAH_00828 | ppnN, queF |
| chr1 | 851748 | 1_1 | Low | Between | GMKMPLAH_00827, GMKMPLAH_00828 | ppnN, queF |
| chr1 | 851748 | 4_2 | Low | Between | GMKMPLAH_00827, GMKMPLAH_00828 | ppnN, queF |
| chr1 | 856777 | 1_1 | Low | Within | GMKMPLAH_00833 | garD_2 |
| chr1 | 856777 | 1_2 | High | Within | GMKMPLAH_00833 | garD_2 |
| chr1 | 856777 | 6_4 | Neg | Within | GMKMPLAH_00833 | garD_2 |
| chr1 | 856777 | 6_3 | Neg | Within | GMKMPLAH_00833 | garD_2 |
| chr1 | 901760 | 1_1 | Low | Within | GMKMPLAH_00870 | narG_1 |
| chr1 | 901760 | 1_2 | High | Within | GMKMPLAH_00870 | narG_1 |
| chr1 | 901760 | 4_2 | Low | Within | GMKMPLAH_00870 | narG_1 |
| chr1 | 910453 | 1_1 | Low | Between | GMKMPLAH_00878, GMKMPLAH_00879 | pncC_2, recA |
| chr1 | 910453 | 1_2 | High | Between | GMKMPLAH_00878, GMKMPLAH_00879 | pncC_2, recA |
| chr1 | 910453 | 6_3 | Neg | Between | GMKMPLAH_00878, GMKMPLAH_00879 | pncC_2, recA |
| chr1 | 912292 | 1_2 | High | Within | GMKMPLAH_00880 | recX |
| chr1 | 918135 | 1_2 | High | Within | GMKMPLAH_00889 | gshA |
| chr1 | 918135 | 1_1 | Low | Within | GMKMPLAH_00889 | gshA |
| chr1 | 918135 | 6_4 | Neg | Within | GMKMPLAH_00889 | gshA |
| chr1 | 918135 | 4_2 | Low | Within | GMKMPLAH_00889 | gshA |
| chr1 | 962581 | 6_5 | Neg | Between | GMKMPLAH_00942, GMKMPLAH_00943 | unknown, xerC_2 |
| chr1 | 975059 | 1_2 | High | Between | GMKMPLAH_00953, GMKMPLAH_00954 | clpB, unknown |
| chr1 | 975059 | 6_4 | Neg | Between | GMKMPLAH_00953, GMKMPLAH_00954 | clpB, unknown |
| chr1 | 989283 | 6_4 | Neg | Within | GMKMPLAH_00965 | emrB_1 |
| chr1 | 992758 | 6_5 | Neg | Within | GMKMPLAH_00968 | unknown |
| chr1 | 992758 | 6_3 | Neg | Within | GMKMPLAH_00968 | unknown |
| chr1 | 1035875 | 6_5 | Neg | Within | MKMPLAH_01010 | rseA |
| chr1 | 1057025 | 1_2 | High | Withi | GMKMPLAH_01030 | hmp |
| chr1 | 1057025 | 1_1 | Low | Within | GMKMPLAH_01030 | hmp |
| chr1 | 1089343 | 1_2 | High | Between | GMKMPLAH_01055, GMKMPLAH_01056 | yfgJ, quiA |
| chr1 | 1154098 | 6_5 | Neg | Within | GMKMPLAH_01111 | amiA |
| chr1 | 1154098 | 1_2 | High | Within | GMKMPLAH_01111 | amiA |
| chr1 | 1154098 | 6_4 | Neg | Within | GMKMPLAH_01111 | amiA |
| chr1 | 1158774 | 6_5 | Neg | Within | GMKMPLAH_01117 | cysW |
| chr1 | 1160400 | 1_2 | High | Within | GMKMPLAH_01119 | cysM |
| chr1 | 1161377 | 1_2 | High | Within | GMKMPLAH_01120 | cpxR_2 |
| chr1 | 1205626 | 1_1 | Low | Within | GMKMPLAH_01170 | unknown |
| chr1 | 1205626 | 1_2 | High | Within | GMKMPLAH_01170 | unknown |
| chr1 | 1216922 | 1_2 | High | Between | GMKMPLAH_01178, GMKMPLAH_01179 | unknown, unknown |
| chr1 | 1216922 | 6_5 | Neg | Between | GMKMPLAH_01178, GMKMPLAH_01179 | unknown, unknown |
| chr1 | 1216922 | 1_1 | Low | Between | GMKMPLAH_01178, GMKMPLAH_01179 | unknown, unknown |
| chr1 | 1231867 | 6_5 | Neg | Within | GMKMPLAH_01188 | unknown |
| chr1 | 1238038 | 1_1 | Low | Between | GMKMPLAH_01194, GMKMPLAH_01195 | unknown, unknown |
| chr1 | 1238038 | 4_2 | High | Between | GMKMPLAH_01194, GMKMPLAH_01195 | unknown, unknown |
| chr1 | 1282696 | 1_2 | High | Within | GMKMPLAH_01241 | yfcF |
| chr1 | 1282696 | 6_5 | Neg | Within | GMKMPLAH_01241 | yfcF |
| chr1 | 1282696 | 1_1 | Low | Within | GMKMPLAH_01241 | yfcF |
| chr1 | 1318195 | 6_5 | Neg | Within | GMKMPLAH_01271 | ubiG |
| chr1 | 1318195 | 6_3 | Neg | Within | GMKMPLAH_01271 | ubiG |
| chr1 | 1323551 | 6_4 | Neg | Within | GMKMPLAH_01273 | rcsC_1 |
| chr1 | 1324532 | 6_5 | Neg | Within | GMKMPLAH_01273 | rcsC_1 |
| chr1 | 1341956 | 1_1 | Low | Within | GMKMPLAH_01277 | unknown |
| chr1 | 1341962 | 1_1 | Low | Within | GMKMPLAH_01277 | unknown |
| chr1 | 1341962 | 1_2 | High | Within | GMKMPLAH_01277 | unknown |
| chr1 | 1341962 | 6_5 | Neg | Within | GMKMPLAH_01277 | unknown |
| chr1 | 1341962 | 6_3 | Neg | Within | GMKMPLAH_01277 | unknown |
| chr1 | 1347555 | 1_1 | Low | Within | GMKMPLAH_01278 | unknown |
| chr1 | 1419363 | 1_2 | High | Between | GMKMPLAH_01341 | unknown |
| chr1 | 1419363 | 1_1 | Low | Between | GMKMPLAH_01341 | unknown |
| chr1 | 1421479 | 1_1 | Low | Within | GMKMPLAH_01343 | sfmC_1 |
| chr1 | 1421479 | 6_3 | Neg | Within | GMKMPLAH_01343 | sfmC_1 |
| chr1 | 1477034 | 1_2 | High | Between | GMKMPLAH_01382 | unknown |
| chr1 | 1477034 | 6_3 | Neg | Between | GMKMPLAH_01382 | unknown |
| chr1 | 1477034 | 1_1 | Low | Between | GMKMPLAH_01382 | unknown |
| chr1 | 1505119 | 1_2 | High | Within | GMKMPLAH_01407 | hisG |
| chr1 | 1510443 | 4_2 | Low | Within | GMKMPLAH_01412 | puuA |
| chr1 | 1510446 | 1_2 | High | Within | GMKMPLAH_01412 | puuA |
| chr1 | 1510446 | 6_4 | Neg | Within | GMKMPLAH_01412 | puuA |
| chr1 | 1531626 | 1_2 | High | Within | GMKMPLAH_01430 | unknown |
| chr1 | 1547393 | 1_2 | High | Within | GMKMPLAH_01445 | unknown |
| chr1 | 1547393 | 1_1 | Low | Within | GMKMPLAH_01445 | unknown |
| chr1 | 1547393 | 6_4 | Neg | Within | GMKMPLAH_01445 | unknown |
| chr1 | 1552888 | 1_2 | High | Within | GMKMPLAH_01451 | argP_2 |
| chr1 | 1552888 | 1_1 | Low | Within | GMKMPLAH_01451 | argP_2 |
| chr1 | 1571212 | 1_2 | High | Within | GMKMPLAH_01467 | davT_1 |
| chr1 | 1571212 | 1_1 | Low | Within | GMKMPLAH_01467 | davT_1 |
| chr1 | 1571212 | 6_4 | Neg | Within | GMKMPLAH_01467 | davT_1 |
| chr1 | 1588518 | 1_2 | High | Within | GMKMPLAH_01480, GMKMPLAH_01481 | unknown, unknown |
| chr1 | 1588518 | 1_1 | Low | Within | GMKMPLAH_01480, GMKMPLAH_01481 | unknown, unknown |
| chr1 | 1592574 | 1_1 | Low | Between | GMKMPLAH_01482, GMKMPLAH_01483 | unknown, unknown |
| chr1 | 1638943 | 1_2 | High | Within | GMKMPLAH_01529 | unknown |
| chr1 | 1639532 | 1_2 | High | Between | GMKMPLAH_01530, GMKMPLAH_01531 | unknown, unknown |
| chr1 | 1639532 | 1_1 | Low | Between | GMKMPLAH_01530, GMKMPLAH_01531 | unknown, unknown |
| chr1 | 1639532 | 6_4 | Neg | Between | GMKMPLAH_01530, GMKMPLAH_01531 | unknown, unknown |
| chr1 | 1651944 | 6_4 | Neg | Within | GMKMPLAH_01545 | fliG |
| chr1 | 1661417 | 6_5 | Neg | Within | GMKMPLAH_01560, GMKMPLAH_01561 | unknown, amyA |
| chr1 | 1661417 | 6_4 | Neg | Within | GMKMPLAH_01560, GMKMPLAH_01561 | unknown, amyA |
| chr1 | 1672392 | 1_2 | High | Within | GMKMPLAH_01567 | unknown |
| chr1 | 1672392 | 1_1 | Low | Within | GMKMPLAH_01567 | unknown |
| chr1 | 1672392 | 6_3 | Neg | Within | GMKMPLAH_01567 | unknown |
| chr1 | 1676703 | 1_2 | High | Within | GMKMPLAH_01570, GMKMPLAH_01571 | fliZ, dcyD |
| chr1 | 1676703 | 4_2 | Low | Within | GMKMPLAH_01570, GMKMPLAH_01571 | fliZ, dcyD |
| chr1 | 1676703 | 1_1 | Low | Within | GMKMPLAH_01570, GMKMPLAH_01571 | fliZ, dcyD |
| chr1 | 1687632 | 1_1 | Low | Within | GMKMPLAH_01578 | putP |
| chr1 | 1704697 | 1_2 | High | Between | GMKMPLAH_01595 | motA |
| chr1 | 1704697 | 6_5 | Neg | Between | GMKMPLAH_01595 | motA |
| chr1 | 1704697 | 1_1 | Low | Between | GMKMPLAH_01595 | motA |
| chr1 | 1726622 | 1_2 | High | Within | GMKMPLAH_01614 | patB |
| chr1 | 1726622 | 1_1 | Low | Within | GMKMPLAH_01614 | patB |
| chr1 | 1726622 | 4_2 | High | Within | GMKMPLAH_01614 | patB |
| chr1 | 1726622 | 6_5 | Neg | Within | GMKMPLAH_01614 | patB |
| chr1 | 1770350 | 6_3 | Neg | Within | GMKMPLAH_01661 | betA |
| chr1 | 1776511 | 1_2 | High | Within | GMKMPLAH_01665 | ycaD_1 |
| chr1 | 1776511 | 1_1 | Low | Within | GMKMPLAH_01665 | ycaD_1 |
| chr1 | 1784493 | 1_2 | High | Within | GMKMPLAH_01671 | msrC |
| chr1 | 1784493 | 1_1 | Low | Within | GMKMPLAH_01671 | msrC |
| chr1 | 1788178 | 1_2 | High | Within | GMKMPLAH_01674 | htpX_1 |
| chr1 | 1860467 | 6_5 | Neg | Within | GMKMPLAH_01747_gene | oppA_1 |
| chr1 | 1860467 | 6_4 | Neg | Within | GMKMPLAH_01747_gene | oppA_1 |
| chr1 | 1886072 | 1_2 | High | Within | GMKMPLAH_01776 | rluB |
| chr1 | 1887090 | 6_5 | Neg | Within | GMKMPLAH_01777 | btuR |
| chr1 | 1888550 | 6_5 | Neg | Within | GMKMPLAH_01779_gene | sohB |
| chr1 | 1928452 | 6_5 | Neg | Within | GMKMPLAH_01812 | sapA |
| chr1 | 1938418 | 1_2 | High | Within | GMKMPLAH_01822 | yfdH |
| chr1 | 1938418 | 1_1 | Low | Within | GMKMPLAH_01822 | yfdH |
| chr1 | 1938418 | 4_2 | Low | Within | GMKMPLAH_01822 | yfdH |
| chr1 | 1938418 | 6_3 | Neg | Within | GMKMPLAH_01822 | yfdH |
| chr1 | 1947094 | 1_2 | High | Within | GMKMPLAH_01831 | ttcA |
| chr1 | 1947094 | 6_4 | Neg | Within | GMKMPLAH_01831 | ttcA |
| chr1 | 1951641 | 1_2 | High | Within | GMKMPLAH_01836 | unknown |
| chr1 | 1951641 | 1_1 | Low | Within | GMKMPLAH_01836 | unknown |
| chr1 | 1951641 | 4_2 | Low | Within | GMKMPLAH_01836 | unknown |
| chr1 | 1951641 | 6_4 | Neg | Within | GMKMPLAH_01836 | unknown |
| chr1 | 1992851 | 1_1 | Low | Within | GMKMPLAH_01868 | aldA |
| chr1 | 1992851 | 4_2 | Low | Within | GMKMPLAH_01868 | aldA |
| chr1 | 1992851 | 6_3 | Neg | Within | GMKMPLAH_01868 | aldA |
| chr1 | 1995771 | 1_2 | High | Within | GMKMPLAH_01872 | rhaT |
| chr1 | 1995771 | 6_5 | Neg | Within | GMKMPLAH_01872 | rhaT |
| chr1 | 1995771 | 4_2 | Low | Within | GMKMPLAH_01872 | rhaT |
| chr1 | 2009079 | 1_1 | Low | Within | GMKMPLAH_01887 | bioD1_1 |
| chr1 | 2009079 | 1_2 | High | Within | GMKMPLAH_01887 | bioD1_1 |
| chr1 | 2009079 | 6_4 | Neg | Within | GMKMPLAH_01887 | bioD1_1 |
| chr1 | 2009079 | 4_2 | Low | Within | GMKMPLAH_01887 | bioD1_1 |
| chr1 | 2015475 | 1_2 | High | Within | GMKMPLAH_01893 | bmr3 |
| chr1 | 2016024 | 4_2 | Low | Within | GMKMPLAH_01893 | bmr3 |
| chr1 | 2037495 | 6_4 | Neg | Within | GMKMPLAH_01912 | unknown |
| chr1 | 2040601 | 1_1 | Low | Within | GMKMPLAH_01914 | potA_1 |
| chr1 | 2040601 | 6_5 | Neg | Within | GMKMPLAH_01914 | potA_1 |
| chr1 | 2040601 | 6_4 | Neg | Within | GMKMPLAH_01914 | potA_1 |
| chr1 | 2040601 | 4_2 | Low | Within | GMKMPLAH_01914 | potA_1 |
| chr1 | 2067877 | 6_5 | Neg | Between | GMKMPLAH_01940 | gcd, unknown |
| chr1 | 2067877 | 1_2 | High | Between | GMKMPLAH_01940 | gcd, unknown |
| chr1 | 2067877 | 1_1 | Low | Between | GMKMPLAH_01940 | gcd, unknown |
| chr1 | 2067877 | 6_4 | Neg | Between | GMKMPLAH_01940 | gcd, unknown |
| chr1 | 2072954 | 1_2 | High | Within | GMKMPLAH_01945 | unknown |
| chr1 | 2072954 | 1_1 | Low | Within | GMKMPLAH_01945 | unknown |
| chr1 | 2088890 | 6_5 | Neg | Within | GMKMPLAH_01963 | dapA_2 |
| chr1 | 2091319 | 6_5 | Neg | Within | GMKMPLAH_01967 | proP_4 |
| chr1 | 2091319 | 6_3 | Neg | Within | GMKMPLAH_01967 | proP_4 |
| chr1 | 2091319 | 6_4 | Neg | Within | GMKMPLAH_01967 | proP_4 |
| chr1 | 2091324 | 1_1 | Low | Within | GMKMPLAH_01967 | proP_4 |
| chr1 | 2091324 | 1_2 | High | Within | GMKMPLAH_01967 | proP_4 |
| chr1 | 2091324 | 6_5 | Neg | Within | GMKMPLAH_01967 | proP_4 |
| chr1 | 2091324 | 6_4 | Neg | Within | GMKMPLAH_01967 | proP_4 |
| chr1 | 2099430 | 1_1 | Low | Within | GMKMPLAH_01978 | unknown |
| chr1 | 2099430 | 1_2 | High | Within | GMKMPLAH_01978 | unknown |
| chr1 | 2099430 | 6_5 | Neg | Within | GMKMPLAH_01978 | unknown |
| chr1 | 2100197 | 6_3 | Neg | Within | GMKMPLAH_01979 | pfkA_2 |
| chr1 | 2107360 | 6_5 | Neg | Between | GMKMPLAH_01985, GMKMPLAH_01986 | pqqB, unknown |
| chr1 | 2112151 | 1_2 | High | Within | GMKMPLAH_01990 | cntO_1 |
| chr1 | 2112238 | 1_2 | High | Within | GMKMPLAH_01990 | cntO_1 |
| chr1 | 2112238 | 6_3 | Neg | Within | GMKMPLAH_01990 | cntO_1 |
| chr1 | 2112238 | 1_1 | Low | Within | GMKMPLAH_01990 | cntO_1 |
| chr1 | 2128507 | 6_5 | Neg | Within | GMKMPLAH_02002 | oqxB26 |
| chr1 | 2160858 | 6_3 | Neg | Between | GMKMPLAH_02030 | rsxG |
| chr1 | 2160858 | 1_1 | Low | Between | GMKMPLAH_02030 | rsxG |
| chr1 | 2160858 | 6_4 | Neg | Between | GMKMPLAH_02030 | rsxG |
| chr1 | 2167879 | 1_2 | High | Between | GMKMPLAH_02037, GMKMPLAH_02038 | pyrG_2, dtpA |
| chr1 | 2167879 | 1_1 | Low | Between | GMKMPLAH_02037, GMKMPLAH_02038 | pyrG_2, dtpA |
| chr1 | 2174712 | 1_2 | High | Between | GMKMPLAH_02046 | unknown |
| chr1 | 2174712 | 6_3 | Neg | Between | GMKMPLAH_02046 | unknown |
| chr1 | 2174712 | 1_1 | Low | Between | GMKMPLAH_02046 | unknown |
| chr1 | 2174712 | 6_4 | Neg | Between | GMKMPLAH_02046 | unknown |
| chr1 | 2178604 | 4_2 | Low | Between | GMKMPLAH_02052 | unknown |
| chr1 | 2197434 | 6_3 | Neg | Within | GMKMPLAH_02067 | glgX_2 |
| chr1 | 2214067 | 1_2 | High | Between | GMKMPLAH_02088, GMKMPLAH_02089 | gloA, rnt |
| chr1 | 2214067 | 4_2 | Low | Between | GMKMPLAH_02088, GMKMPLAH_02089 | gloA, rnt |
| chr1 | 2215127 | 1_2 | High | Between | GMKMPLAH_02090, GMKMPLAH_02091 | grxD, mepH |
| chr1 | 2215127 | 6_5 | Neg | Between | GMKMPLAH_02090, GMKMPLAH_02091 | grxD, mepH |
| chr1 | 2215127 | 6_3 | Neg | Between | GMKMPLAH_02090, GMKMPLAH_02091 | grxD, mepH |
| chr1 | 2215127 | 4_2 | Low | Between | GMKMPLAH_02090, GMKMPLAH_02091 | grxD, mepH |
| chr1 | 2239386 | 6_3 | Neg | Between | GMKMPLAH_02114 | ppsA |
| chr1 | 2245727 | 6_5 | Neg | Within | GMKMPLAH_02118 | ydiV |
| chr1 | 2257306 | 1_2 | High | Between | GMKMPLAH_02130 | unknown |
| chr1 | 2257306 | 1_1 | Low | Between | GMKMPLAH_02130 | unknown |
| chr1 | 2266426 | 6_5 | Neg | Between | GMKMPLAH_02141 | bcr_2 |
| chr1 | 2300762 | 6_5 | Neg | Within | GMKMPLAH_02167 | norW |
| chr1 | 2311243 | 1_1 | Low | Within | GMKMPLAH_02175 | unknown |
| chr1 | 2311243 | 1_2 | High | Within | GMKMPLAH_02175 | unknown |
| chr1 | 2311243 | 4_2 | Low | Within | GMKMPLAH_02175 | unknown |
| chr1 | 2317282 | 1_1 | Low | Within | GMKMPLAH_02181, GMKMPLAH_02182 | chaB, chaA |
| chr1 | 2317282 | 1_2 | High | Between | GMKMPLAH_02181, GMKMPLAH_02182 | chaB, chaA |
| chr1 | 2319083 | 6_4 | Neg | Within | GMKMPLAH_02183 | kdsA |
| chr1 | 2359889 | 1_2 | High | Within | GMKMPLAH_02220 | unknown |
| chr1 | 2359889 | 1_1 | Low | Within | GMKMPLAH_02220 | unknown |
| chr1 | 2359889 | 6_4 | Neg | Within | GMKMPLAH_02220 | unknown |
| chr1 | 2359889 | 6_5 | Neg | Within | GMKMPLAH_02220 | unknown |
| chr1 | 2393791 | 1_2 | High | Between | GMKMPLAH_02250 | eamA_2 |
| chr1 | 2393791 | 6_5 | Neg | Between | GMKMPLAH_02250 | eamA_2 |
| chr1 | 2393791 | 6_3 | Neg | Between | GMKMPLAH_02250 | eamA_2 |
| chr1 | 2393791 | 1_1 | Low | Between | GMKMPLAH_02250 | eamA_2 |
| chr1 | 2421873 | 1_2 | High | Between | GMKMPLAH_02280 | unknown |
| chr1 | 2421873 | 1_1 | Low | Between | GMKMPLAH_02280 | unknown |
| chr1 | 2421873 | 6_4 | Neg | Between | GMKMPLAH_02280 | unknown |
| chr1 | 2432766 | 6_4 | Neg | Within | GMKMPLAH_02293 | phoQ |
| chr1 | 2436834 | 1_1 | Low | Within | GMKMPLAH_02296 | cobB |
| chr1 | 2436834 | 6_4 | Neg | Within | GMKMPLAH_02296 | cobB |
| chr1 | 2436834 | 6_5 | Neg | Within | GMKMPLAH_02296 | cobB |
| chr1 | 2436834 | 4_2 | Low | Within | GMKMPLAH_02296 | cobB |
| chr1 | 2442458 | 6_5 | Neg | Within | GMKMPLAH_02301 | mfd |
| chr1 | 2454701 | 1_2 | High | Between | GMKMPLAH_02312 | ptsG |
| chr1 | 2454701 | 1_1 | Low | Between | GMKMPLAH_02312 | ptsG |
| chr1 | 2463163 | 6_4 | Neg | Within | GMKMPLAH_02321 | fabD_2 |
| chr1 | 2463163 | 6_5 | Neg | Within | GMKMPLAH_02321 | fabD_2 |
| chr1 | 2468165 | 1_2 | High | Within | GMKMPLAH_02321 | fabD_2 |
| chr1 | 2474246 | 1_2 | High | Within | GMKMPLAH_02330 | flgK |
| chr1 | 2474246 | 6_5 | Neg | Within | GMKMPLAH_02330 | flgK |
| chr1 | 2504491 | 6_4 | Neg | Within | GMKMPLAH_02363 | clsC |
| chr1 | 2507494 | 1_2 | High | Within | GMKMPLAH_02366 | unknown |
| chr1 | 2507494 | 1_1 | Low | Within | GMKMPLAH_02366 | unknown |
| chr1 | 2507494 | 6_5 | Neg | Within | GMKMPLAH_02366 | unknown |
| chr1 | 2535328 | 1_2 | High | Between | GMKMPLAH_02399 | unknown |
| chr1 | 2535328 | 6_3 | Neg | Between | GMKMPLAH_02399 | unknown |
| chr1 | 2535328 | 1_1 | Low | Between | GMKMPLAH_02399 | unknown |
| chr1 | 2535710 | 6_4 | Neg | Within | GMKMPLAH_02399 | unknown |
| chr1 | 2567103 | 6_4 | Neg | Within | GMKMPLAH_02437 | unknown |
| chr1 | 2590187 | 1_2 | High | Within | GMKMPLAH_02472 | rutF |
| chr1 | 2590187 | 1_1 | Low | Within | GMKMPLAH_02472 | rutF |
| chr1 | 2590187 | 6_4 | Neg | Within | GMKMPLAH_02472 | rutF |
| chr1 | 2591694 | 6_3 | Neg | Within | GMKMPLAH_02473 | unknown |
| chr1 | 2595438 | 1_1 | Low | Within | GMKMPLAH_02478 | unknown |
| chr1 | 2602012 | 6_4 | Neg | Within | GMKMPLAH_02488 | mgsA |
| chr1 | 2610482 | 6_5 | Neg | Within | GMKMPLAH_02495 | matP |
| chr1 | 2627354 | 1_2 | High | Within | GMKMPLAH_02508 | ssuE |
| chr1 | 2627354 | 6_3 | Neg | Within | GMKMPLAH_02508 | ssuE |
| chr1 | 2654595 | 6_3 | Neg | Within | GMKMPLAH_02529 | unknown |
| chr1 | 2688059 | 1_2 | High | Within | GMKMPLAH_02553 | clpA |
| chr1 | 2688059 | 1_1 | Low | Within | GMKMPLAH_02553 | clpA |
| chr1 | 2695927 | 1_1 | Low | Within | GMKMPLAH_02559 | ltaE |
| chr1 | 2715662 | 4_2 | Low | Within | GMKMPLAH_02582 | dacC |
| chr1 | 2720452 | 1_1 | Low | Within | GMKMPLAH_02587 | gsiD_3 |
| chr1 | 2779388 | 1_2 | High | Within | GMKMPLAH_02640 | unknown |
| chr1 | 2779388 | 1_1 | Low | Within | GMKMPLAH_02640 | unknown |
| chr1 | 2779388 | 4_2 | Low | Within | GMKMPLAH_02640 | unknown |
| chr1 | 2779388 | 6_4 | Neg | Within | GMKMPLAH_02640 | unknown |
| chr1 | 2791789 | 1_2 | High | Within | GMKMPLAH_02646 | pbpG_2 |
| chr1 | 2791789 | 1_1 | Low | Within | GMKMPLAH_02646 | pbpG_2 |
| chr1 | 2791789 | 4_2 | Low | Within | GMKMPLAH_02646 | pbpG_2 |
| chr1 | 2792369 | 6_5 | Neg | Within | GMKMPLAH_02647 | yohC |
| chr1 | 2832504 | 4_2 | Low | Within | GMKMPLAH_02686 | galM |
| chr1 | 2857363 | 6_5 | Neg | Within | GMKMPLAH_02713 | sucB |
| chr1 | 2869931 | 1_2 | High | Within | GMKMPLAH_02724 | pxpA |
| chr1 | 2869931 | 1_1 | Low | Within | GMKMPLAH_02724 | pxpA |
| chr1 | 2891017 | 6_5 | Neg | Within | GMKMPLAH_02745 | asnB |
| chr1 | 2891017 | 6_4 | Neg | Within | GMKMPLAH_02745 | asnB |
| chr1 | 2892953 | 4_2 | Low | Between | GMKMPLAH_02746, GMKMPLAH_02747 | unknown, unknown |
| chr1 | 2892953 | 1_1 | Low | Between | GMKMPLAH_02746, GMKMPLAH_02747 | unknown, unknown |
| chr1 | 2892953 | 6_5 | Neg | Between | GMKMPLAH_02746, GMKMPLAH_02747 | unknown, unknown |
| chr1 | 2904975 | 1_2 | High | Between | GMKMPLAH_02763, GMKMPLAH_02764 | unknown, unknown |
| chr1 | 2926322 | 1_2 | High | Between | GMKMPLAH_02785, GMKMPLAH_02786 | unknown, gntT |
| chr1 | 2926322 | 1_1 | Low | Between | GMKMPLAH_02785, GMKMPLAH_02786 | unknown, gntT |
| chr1 | 2926917 | 6_5 | Neg | Between | GMKMPLAH_02785, GMKMPLAH_02786 | unknown, gntT |
| chr1 | 2939545 | 4_2 | Low | Within | GMKMPLAH_02798 | unknown |
| chr1 | 2939545 | 1_2 | High | Within | GMKMPLAH_02798 | unknown |
| chr1 | 2957183 | 6_3 | Neg | Within | GMKMPLAH_02818 | unknown |
| chr1 | 2957183 | 6_4 | Neg | Within | GMKMPLAH_02818 | unknown |
| chr1 | 2976848 | 1_1 | Low | Within | MKMPLAH_02839 | ybaK |
| chr1 | 2976848 | 1_2 | High | Within | MKMPLAH_02839 | ybaK |
| chr1 | 2992262 | 1_2 | High | Within | GMKMPLAH_02852 | dnaX |
| chr1 | 2992262 | 1_1 | Low | Within | GMKMPLAH_02852 | dnaX |
| chr1 | 3000588 | 1_2 | High | Within | GMKMPLAH_02860 | acrB_2 |
| chr1 | 3000690 | 1_1 | Low | Between | GMKMPLAH_02860, GMKMPLAH_02861 | acrB_2, rpmE2 |
| chr1 | 3005890 | 6_5 | Neg | Within | GMKMPLAH_02870 | ybaY |
| chr1 | 3005890 | 6_4 | Neg | Within | GMKMPLAH_02870 | ybaY |
| chr1 | 3005890 | 6_3 | Neg | Within | GMKMPLAH_02870 | ybaY |
| chr1 | 3005890 | 4_2 | Low | Within | GMKMPLAH_02870 | ybaY |
| chr1 | 3029375 | 6_5 | Neg | Within | GMKMPLAH_02889 | unknown |
| chr1 | 3029375 | 6_4 | Neg | Within | GMKMPLAH_02889 | unknown |
| chr1 | 3029375 | 6_3 | Neg | Within | GMKMPLAH_02889 | unknown |
| chr1 | 3078181 | 4_2 | Low | Within | GMKMPLAH_02936 | secF |
| chr1 | 3120151 | 1_2 | High | Between | GMKMPLAH_02975 | unknown |
| chr1 | 3120151 | 1_1 | Low | Between | GMKMPLAH_02975 | unknown |
| chr1 | 3122981 | 1_2 | High | Within | GMKMPLAH_02976 | unknown |
| chr1 | 3122981 | 1_1 | Low | Within | GMKMPLAH_02976 | unknown |
| chr1 | 3122981 | 6_4 | Neg | Within | GMKMPLAH_02976 | unknown |
| chr1 | 3122981 | 6_3 | Neg | Within | GMKMPLAH_02976 | unknown |
| chr1 | 3133837 | 4_2 | Low | Between | GMKMPLAH_02989 | dbpA |
| chr1 | 3153009 | 1_2 | High | Within | GMKMPLAH_03008 | mglA_4 |
| chr1 | 3153009 | 1_1 | Low | Within | GMKMPLAH_03008 | mglA_4 |
| chr1 | 3168943 | 1_2 | High | Between | GMKMPLAH_03022 | yafV |
| chr1 | 3168943 | 1_1 | Low | Between | GMKMPLAH_03022 | yafV |
| chr1 | 3168943 | 6_3 | Neg | Between | GMKMPLAH_03022 | yafV |
| chr1 | 3173680 | 6_5 | Neg | Within | GMKMPLAH_03028 | yecS_2 |
| chr1 | 3236663 | 1_2 | High | Within | GMKMPLAH_03090 | dgt |
| chr1 | 3236663 | 1_1 | Low | Within | GMKMPLAH_03090 | dgt |
| chr1 | 3269348 | 1_1 | Low | Within | GMKMPLAH_03120 | glcR_1 |
| chr1 | 3283784 | 1_2 | High | Within | GMKMPLAH_03133 | lpdA |
| chr1 | 3283784 | 1_1 | Low | Within | GMKMPLAH_03133 | lpdA |
| chr1 | 3283784 | 6_3 | Neg | Within | GMKMPLAH_03133 | lpdA |
| chr1 | 3283784 | 6_4 | Neg | Within | GMKMPLAH_03133 | lpdA |
| chr1 | 3316656 | 1_1 | Low | Within | GMKMPLAH_03160 | ftsW |
| chr1 | 3378768 | 1_2 | High | Between | GMKMPLAH_03208 | proP_4 |
| chr1 | 3378768 | 1_1 | Low | Between | GMKMPLAH_03208 | proP_4 |
| chr1 | 3388106 | 6_4 | Neg | Within | GMKMPLAH_03215 | trmL_2 |
| chr1 | 3429836 | 4_2 | Low | Within | GMKMPLAH_03255 | ydfJ |
| chr1 | 3450562 | 1_2 | High | Within | GMKMPLAH_03268 | unknown |
| chr1 | 3450562 | 1_1 | Low | Within | GMKMPLAH_03268 | unknown |
| chr1 | 3450562 | 6_3 | Neg | Within | GMKMPLAH_03268 | unknown |
| chr1 | 3464499 | 1_1 | Low | Within | GMKMPLAH_03276 | yajL_2 |
| chr1 | 3464499 | 6_5 | Neg | Within | GMKMPLAH_03276 | yajL_2 |
| chr1 | 3464499 | 6_4 | Neg | Within | GMKMPLAH_03276 | yajL_2 |
| chr1 | 3467577 | 1_2 | High | Within | GMKMPLAH_03279_ | unknown |
| chr1 | 3467577 | 1_1 | Low | Within | GMKMPLAH_03279_ | unknown |
| chr1 | 3467577 | 4_2 | Low | Within | GMKMPLAH_03279_ | unknown |
| chr1 | 3476436 | 1_2 | High | Within | GMKMPLAH_03284 | unknown |
| chr1 | 3476436 | 1_1 | Low | Within | GMKMPLAH_03284 | unknown |
| chr1 | 3518009 | 4_2 | Low | Between | GMKMPLAH_03317 | dorA |
| chr1 | 3553647 | 1_1 | Low | Between | GMKMPLAH_03350 | greA |
| chr1 | 3553647 | 1_2 | High | Between | GMKMPLAH_03350 | greA |
| chr1 | 3553647 | 6_3 | Neg | Between | GMKMPLAH_03350 | greA |
| chr1 | 3562013 | 1_2 | High | Within | GMKMPLAH_03360 | mlaC |
| chr1 | 3562013 | 4_2 | Low | Within | GMKMPLAH_03360 | mlaC |
| chr1 | 3562013 | 6_4 | Neg | Within | GMKMPLAH_03360 | mlaC |
| chr1 | 3573671 | 1_1 | Low | Between | GMKMPLAH_03375, GMKMPLAH_03376 | mtgA, acrB |
| chr1 | 3573671 | 1_2 | High | Between | GMKMPLAH_03375, GMKMPLAH_03376 | mtgA, acrB |
| chr1 | 3573671 | 4_2 | Low | Between | GMKMPLAH_03375, GMKMPLAH_03376 | mtgA, acrB |
| chr1 | 3573671 | 6_4 | Neg | Between | GMKMPLAH_03375, GMKMPLAH_03376 | mtgA, acrB |
| chr1 | 3573745 | 1_1 | Low | Between | GMKMPLAH_03375, GMKMPLAH_03376 | mtgA, acrB |
| chr1 | 3573745 | 6_5 | Neg | Between | GMKMPLAH_03375, GMKMPLAH_03376 | mtgA, acrB |
| chr1 | 3573745 | 1_2 | High | Between | GMKMPLAH_03375, GMKMPLAH_03376 | mtgA, acrB |
| chr1 | 3581634 | 1_1 | Low | Within | GMKMPLAH_03377 | unknown |
| chr1 | 3588972 | 1_2 | High | Within | GMKMPLAH_03384 | yhcB |
| chr1 | 3588972 | 1_1 | Low | Within | GMKMPLAH_03384 | yhcB |
| chr1 | 3588972 | 6_3 | Neg | Within | GMKMPLAH_03384 | yhcB |
| chr1 | 3588972 | 6_4 | Neg | Within | GMKMPLAH_03384 | yhcB |
| chr1 | 3601265 | 1_2 | High | Within | GMKMPLAH_03398 | dcuA |
| chr1 | 3601265 | 6_5 | Neg | Within | GMKMPLAH_03398 | dcuA |
| chr1 | 3613778 | 1_1 | Low | Within | GMKMPLAH_03411 | dmlR_8 |
| chr1 | 3662169 | 6_4 | Neg | Within | GMKMPLAH_03449 | gltP |
| chr1 | 3662308 | 1_1 | Low | Within | GMKMPLAH_03449 | gltP |
| chr1 | 3662308 | 6_5 | Neg | Within | GMKMPLAH_03449 | gltP |
| chr1 | 3662308 | 6_3 | Neg | Within | GMKMPLAH_03449 | gltP |
| chr1 | 3662308 | 6_4 | Neg | Within | GMKMPLAH_03449 | gltP |
| chr1 | 3662308 | 4_2 | Low | Within | GMKMPLAH_03449 | gltP |
| chr1 | 3679247 | 6_4 | Neg | Within | GMKMPLAH_03465, GMKMPLAH_03466 | unknown, unknown |
| chr1 | 3694513 | 1_1 | Low | Between | GMKMPLAH_03480 | dusA |
| chr1 | 3698696 | 1_2 | High | Within | BBIKNCBD_03556 | dinF_2 |
| chr1 | 3698696 | 1_1 | Low | Within | BBIKNCBD_03556 | dinF_2 |
| chr1 | 3711119 | 1_2 | High | Within | GMKMPLAH_03497 | unknown |
| chr1 | 3711119 | 1_1 | Low | Within | GMKMPLAH_03497 | unknown |
| chr1 | 3711119 | 6_4 | Neg | Within | GMKMPLAH_03497 | unknown |
| chr1 | 3739860 | 1_1 | Low | Within | GMKMPLAH_03515 | purH |
| chr1 | 3739860 | 6_3 | Neg | Within | GMKMPLAH_03515 | purH |
| chr1 | 3739860 | 6_4 | Neg | Within | GMKMPLAH_03515 | purH |
| chr1 | 3739860 | 4_2 | High | Within | GMKMPLAH_03515 | purH |
| chr1 | 3759956 | 6_5 | Neg | Within | GMKMPLAH_03515 | purH |
| chr1 | 3770111 | 1_2 | High | Within | GMKMPLAH_03546 | unknown |
| chr1 | 3770111 | 1_1 | Low | Within | GMKMPLAH_03546 | unknown |
| chr1 | 3804680 | 1_2 | High | Within | GMKMPLAH_03576 | pldB |
| chr1 | 3804680 | 1_1 | Low | Within | GMKMPLAH_03576 | pldB |
| chr1 | 3804680 | 6_5 | Neg | Within | GMKMPLAH_03576 | pldB |
| chr1 | 3816593 | 1_2 | High | Between | GMKMPLAH_03588 | yigB |
